# Screening and Analysis of Multiclass Veterinary Drug Residues in Animal Source Foods using UPLC-Q-Exactive Orbitrap/MS

**DOI:** 10.1007/s00128-021-03273-w

**Published:** 2021-06-07

**Authors:** Wentao Zhao, Rui Jiang, Wenping Guo, Chao Guo, Shilei Li, Juanqiang Wang, Shouwei Wang, Yingying Li

**Affiliations:** China Meat Research Center, 100068 Beijing, People’s Republic of China

**Keywords:** UPLC-Q-Exactive Orbitrap/MS, Veterinary drug residues, Animal source foods, Qualitative screening, Quantitative determination

## Abstract

A rapid, simple, and sensitive method of detecting veterinary drug residues in animal food sources, including poultry and pork, was developed and validated. The method was optimized for over 155 veterinary drugs of 21 different classes. Sample pretreatment included a simple solid-liquid extraction step with 0.2% formic acid-acetonitrile-water and a purification step with a PRiME HLB (hydrophile-lipophile balance) solid-phase extraction cartridge. Data were collected using ultra-high-performance liquid chromatography coupled to Quadrupole-Exactive Orbitrap mass spectrometry. The limits of detection of 155 veterinary drugs ranged from 0.1 µg/kg to 10 µg/kg. The recovery rates were between 79.2 and 118.5 % in all matrices studied, with relative standard deviation values less than 15% (n = 6). The evaluated method allows the reliable screening, quantification, and identification of 155 veterinary drug residues in animal source food and has been successfully applied in authentic samples.

**Supplementary Information:**

The online version of this article (10.1007/s00128-021-03273-w) contains supplementary material, which is available to authorized users.

Meat is an indispensable part of the human diet (Laranjo et al. 2017). During breeding, animals are supplemented with veterinary drugs to treat and prevent diseases (Kpodékon et al. 2015; Knap 2020). However, excessive use and misuse of veterinary drugs can lead to human health risks and environmental pollution (Liu et al. 2019; Trishna et al. 2018). In recent years, food contamination by veterinary drug residue has become public health concern worldwide (Beyene 2016; Organization 2016; Song et al. 2016). Hence, the maximum residue limits of veterinary drugs in food products must be set (Sanders et al. 2016; Tang et al. 2017).

Veterinary drugs differ in type and chemical properties; thus, sample pretreatment and analytical techniques also differ (Moretti et al. 2016; Mainero et al. 2017). At present, analytical techniques used for veterinary drug residues in animal food sources are mainly based on HPLC (Kang et al. 2014; Samanidou et al. 2015), UPLC-MS/MS (Taylor et al. 2019; Zhao et al. 2018), UPLC-QE (Jia et al. 2017; Wang et al. 2018a), UPLC-QTOF (Wang et al. 2018b; Kang et al. 2017), and GC-MS (Xue et al. 2017; Lozano et al. 2019). HPLC is inappropriate for the simultaneous detection of multiple residues of animal origin, and it can only be used for qualitative discrimination (Anumol et al. 2017). GC-MS is unsuitable for the detection of volatile, highly polar, and pyrolytic compounds. The most commonly used methods for detecting multi-residues in animal food sources include UPLC-MS/MS, UPLC-QE, and UPLC-QTOF (Xu et al. 2018). Steven used UPLC-MS/MS to detect 62 veterinary drug residues in the bovine kidney, with a recovery rate of 70% and a repeatability of less than 25% (Steven et al. 2012). Of the residues tested, 50 drugs met the qualitative confirmation criteria. No quantitative analysis was conducted in this method. Xie used HPLC-MS/MS to determine 54 veterinary drug residues in pork, belonging to classes of sulfonamides and quinolones. The method quantification limits range from 0.3 g/kg to 3.0 g/kg, and the recovery rates range from 20.9% to 121.0% (Xie et al. 2012).

UPLC-MS/MS has insufficient anti-interference ability in a complex matrix and is inaccurate for the qualitative analysis of multi-residues in animal food sources. Compared with UPLC-MS/MS, high-resolution MS has better qualitative ability and can effectively avoid false-negative and false-positive results.

High-resolution MS can be coupled to UPLC-QTOF and UPLC Q-Exactive, both of which determine the precise mass number of target compounds but differ in detection principle. Wang used UPLC-QTOF to determine the residues of aminoglycosides, β-lactams, and other veterinary drugs in milk and honey samples. This method can be used for the quantitative analysis of 31 and 34 compounds in honey and milk samples and conductand qualitative analysis of 54 and 34 compounds in honey and milk samples, respectively (Wang et al. 2012). Wang screened and quantified 125 veterinary drug residues in milk by using UPLC-Exactive-Orbitrap, with limits of quantification (LOQ) as low as 1.0 µg/kg (Wang et al. 2015).

This study aims to use UPLC Orbitrap Q-Exactive HF-X as a rapid and accurate qualitative and quantitative method for detecting veterinary drug residues belonging to 21 classes in foods, such as pork, poultry meat, and their innards with simple pretreatment. Qualitative and quantitative results proved to be accurate, and the method was applied to authentic samples for the rapid detection of veterinary drug residues.

## Methods and Materials

HPLC-grade methanol, acetonitrile (ACN), formic acid (FA), acetic acid, and ammonium acetate were procured from Thermo Fisher (USA). All veterinary drugs were obtained from Sigma-Aldrich (Germany).

Standard substances (10.0 mg) were weighed and placed in a 10 mL brown volumetric flask. They were dissolved, volume-stabilized with appropriate reagents, such as methanol and ACN 1.0 mg/mL standard reserve solutions, and then stored at − 18°C. The standard reserve solution prepared with water was stored at 4°C. Intermediate veterinary drug standard mix working solution was prepared as 10.0 µg/mL in methanol from stock solutions.

ACN (800 mL), water (198 mL), and FA (2 mL) were mixed to form a solution of ACN/water/FA (80 + 19.8 + 0.2, v/v). Water/FA (99.9 + 0.1, v/v) was prepared by mixing 999 mL of water with 1 mL of FA.

The samples were collected from abattoirs, supermarkets, or farmers’ markets. A 2.0 g sample was weighed into a 50 mL centrifuge tube. Then, 10 mL of ACN–FA solution was added into the sample, which was shaken on a mechanical shaker. After 30 min, the sample mixture was centrifuged at 4000 r/min for 3 min in a centrifuge. The supernatant (5 mL) was transferred into a PRiME HLB SPE column. The effluent was collected and blow-dried under nitrogen. The extracts were diluted up to 0.5 mL with water/FA (99.9 + 0.1, v/v) and vortexed for 30 s. Sample extracts were analyzed using a UPLC-Q-Exactive Orbitrap/MS system.

A Thermo Scientific Q-Exactive HF-X was used to detect veterinary drugs. Veterinary drug residue analysis was performed with a Thermo Scientific Vanquish UHPLC, and MS analyses were conducted using Thermo Scientific Q-Exactive HF coupled to a Nanospray Flex ion source. The extract ion chromatograms of 155 veterinary drugs were shown in supporting information (Fig. S1).

In the positive mode, the mobile phase A was 0.1 % FA/H_2_O; in the negative mode, the mobile phase A was 5 mM ammonium acetate and the mobile phase B was ACN. The gradient profile was as follows: 0 min: 98% A; 0–20 min: 98%–0% A; 20–25 min: 0% A; 25–28 min: 98% A. The flow rate was 0.5 mL/min, and the injection volume was 5 µL. The UPLC column was an Agilent Eclipse plus C18, 3.0 × 150 mm, 1.8 μm. The column oven temperature was set at 40°C, and the auto-sampler temperature was set at 4°C.

The QE MS parameters were as follows: ScanType, Full MS-dd-MS2; Polarity, Negative; Sheath gas flow rate, 40 L; Aux gas flow rate, 15 L; Spray voltage, 3.5 KV; Capillary temp, 325 °C; Aux gas heater temp, 450°C; Full MS Resolution, 60 000; dd-MS2 Resolution, 15 000. Quantitatively method, the exact ratio of the parent ion and the characteristic fragment ion of the target compound; qualitatively method, the peak area of parent ion.

The calibration curves were validated over the concentration range 1 × LOD – 25 × LOD for veterinary drug residues in animal source foods. The correlation coefficients of all the curves were greater than 0.99, and the deviations of the back-calculated concentrations from their nominal values were within ± 15%. Results were fitted to linear regression analysis using 1/x^2^ as the weighting factor.

With the peak area of the standard substance as the y-axis and the corresponding working liquid concentration (ng/mL) as the x-axis, all veterinary drugs showed good linearity in their appropriate concentration range. Recovery is a function of additive concentration, and a signal-to-noise ratio of 3 is acceptable to estimate the detection limit of the method (see Table 1). In total, 155 types of veterinary drug standard solutions were added to the negative samples, and the LOD and LOQ were calculated by taking three times the standard deviation for LOD and 6 or 10 times the standard deviation for LOQ. Six samples were measured in parallel with each standard level. As shown in Table 1, the average recovery rate of 155 veterinary drugs was between 79.2% and 118.5%, RSD ≤ 15 %.

**Table 1:** Linear range, detection limit, standard recovery, and relative standard deviation of 155 veterinary drug residues in pork samples (n = 6)

No	Compound	Linearity range ng/mL	LOD µg/kg	Spiked 3 × LOD		Spiked 5 × LOD		Spiked 10 × LOD	
				R/%	RSD/%	R/%	RSD/%	R/%	RSD/%
1	Sulfaphenazole	10–250	5.0	86.6	6.3	83.5	8.1	88.3	9.4
2	Sulfabenzamide	10–250	5.0	94.8	5.9	95.1	1.3	93.9	3.0
3	Sulfapyridine	10–250	5.0	104.4	3.4	93.7	3.6	90.4	6.7
4	Sulfacetamide	10–250	5.0	101.1	7.8	94.0	14.1	108.3	9.6
5	Sulfameter	10–250	5.0	100.5	4.7	96.1	2.8	83.6	2.7
6	Sulfamoxole	10–250	5.0	89.3	6.8	103.3	4.3	101.0	2.3
7	Sulfisoxazole	10–250	5.0	93.6	5.3	106.9	5.0	107.0	3.7
8	Sulfamethazine	10–250	5.0	86.2	8.2	100.8	5.6	80.8	10.3
9	Sulfisomidine	10–250	5.0	83.3	7.9	83.4	10.9	96.3	5.2
10	Sulfamethoxazole	10–250	5.0	92.3	9.9	95.0	11.5	102.6	13.6
11	Sulfamerazine	10–250	5.0	99.4	11.3	91.4	8.9	107.5	11.9
12	Sulfamethoxypyridazine	10–250	5.0	93.6	7.5	101.1	10.8	90.1	6.2
13	Sulfadimethoxypyrimidine	10–250	5.0	98.2	5.4	108.3	10.5	91.6	8.9
14	Sulfamonnomethoxine	10–250	5.0	91.8	3.8	90.4	7.9	92.4	9.3
15	Sulfaquinoxaline	10–250	5.0	86.2	6.7	80.8	12.8	84.1	12.0
16	Sulfadimoxine	10–250	5.0	89.0	9.1	98.9	7.3	98.5	5.7
17	Sulfaclozine	10–250	5.0	118.5	7.4	111.1	11.4	115.3	14.3
18	Sulfachloropyridazine	10–250	5.0	89.7	2.5	95.6	3.7	87.1	4.5
19	Sulfaguanidine	10–250	5.0	109.1	6.7	106.7	13.4	103.0	7.0
20	Sulfadiazine	10–250	5.0	95.7	5.8	93.0	9.2	90.5	0.6
21	Sulfathiazole	10–250	5.0	106.4	4.4	105.9	0.8	96.9	5.5
22	Sulfamethizole	10–250	5.0	98.5	7.1	105.9	8.9	107.0	9.9
23	Orbifloxacin	6–150	3.0	114.0	8.3	115.9	5.8	100.5	13.8
24	Danofloxacin	6–150	3.0	111.9	12.6	99.0	9.2	93.0	14.2
25	Enrofloxacin	10–250	5.0	107.1	10.5	93.5	9.0	110.5	4.9
26	Flumequine	6–150	3.0	111.3	4.4	98.3	4.3	108.6	5.4
27	Fleroxacin	6–150	3.0	88.7	7.1	108.1	3.8	90.5	2.5
28	Ciprofloxacin	6–150	3.0	88.0	1.5	99.9	2.8	101.5	3.4
29	Lomefloxacin	6–150	3.0	103.1	12.0	90.8	9.0	91.2	5.4
30	Nalidixic acid	6–150	3.0	88.3	4.8	91.2	8.7	90.5	2.4
31	Norfloxacin	6–150	3.0	101.0	12.9	113.1	6.7	104.0	6.9
32	Pefloxacin	6–150	3.0	103.8	3.4	97.5	5.2	99.1	7.9
33	Sarafloxacin	6–150	3.0	86.4	12.2	85.6	14.6	82.5	7.9
34	Difloxacin	6–150	3.0	112.4	8.3	107.4	11.5	102.7	12.7
35	Sparfloxacin	6–150	3.0	80.5	6.5	92.4	7.0	86.9	4.7
36	Enoxacin	10–250	5.0	101.0	2.4	107.9	7.3	108.1	5.1
37	Ofloxacin	6–150	3.0	98.4	12.2	90.8	6.1	86.1	10.9
38	Clenbuterol	1–25	0.5	92.7	11.0	85.0	7.3	104.2	12.0
39	Ractopamine	1–25	0.5	88.7	6.5	86.2	4.4	85.5	7.3
40	Clorprenaline	1–25	0.5	101.8	11.9	106.2	12.5	108.1	10.5
41	Penbutolol	1–25	0.5	80.3	3.1	79.7	7.5	85.0	6.5
42	Metaproterenol	1–25	0.5	106.5	12.3	107.1	10.2	98.6	10.9
43	Formoterol	1–25	0.5	101.3	6.1	104.2	4.6	86.8	15.2
44	Fenoterol	1–25	0.5	93.3	8.6	93.8	11.1	91.7	9.5
45	Cimbuterol	1–25	0.5	101.7	8.0	95.9	14.3	97.8	4.8
46	Bambuterol	1–25	0.5	87.4	4.1	94.6	8.7	109.0	8.7
47	Phenylethanolamine A	1–25	0.5	84.6	9.2	104.0	9.3	101.4	7.2
48	Thiabendazole	1–25	0.5	86.5	6.7	110.6	12.9	103.8	6.4
49	Salbutamol	1–25	0.5	108.4	9.6	93.1	11.9	101.3	12.7
50	Terbutaline	2–50	1.0	107.6	10.7	96.8	8.5	93.1	6.4
51	Tulobuterol	1–25	0.5	114.4	1.8	102.8	4.4	118.3	3.8
52	Cimaterol	1–25	0.5	104.6	9.2	111.7	6.2	95.8	6.4
53	Florfenicol	10–250	5.0	105.3	14.9	103.6	7.3	116.2	11.5
54	Chlortetracycline	10–250	5.0	98.6	11.4	104.0	4.5	100.7	8.7
55	Doxycycline	10–250	5.0	99.5	9.9	95.5	10.1	99.0	11.4
56	Tetracycline	10–250	5.0	93.2	4.9	105.0	7.5	103.0	13.0
57	Oxytetracycline	10–250	5.0	102.8	8.4	94.3	10.8	98.1	10.9
58	Erythromycin	2–50	1.0	80.1	3.0	86.7	6.6	79.9	6.4
59	Kitasamycin	2–50	1.0	91.1	12.9	84.2	14.6	95.0	5.5
60	Lincomycin	2–50	1.0	105.6	3.8	111.7	12.1	102.5	10.4
61	Tylosin	2–50	1.0	98.7	9.7	112.1	14.0	109.7	8.6
62	Tilmicosin	2–50	1.0	107.3	8.4	116.7	8.4	118.2	9.3
63	Tylosin 3-acetate 4B-(3-methylbutanoate) (2R,3R)-2,3-dihydroxybutanedioate	2–50	1.0	117.0	7.6	118.5	11.1	114.2	9.5
64	Oleandomycin	2–50	1.0	107.3	4.6	106.8	6.6	102.6	3.7
65	Ronidazole	2–50	1.0	99.7	3.6	92.7	5.1	101.8	3.4
66	Dimetridazole	2–50	1.0	111.3	8.4	104.9	7.8	108.8	5.3
67	Metronidazole-hydroxy	2–50	1.0	96.0	14.3	111.9	8.3	112.5	10.2
68	Dimetridazolr-hydroxy	2–50	1.0	97.2	11.6	105.7	9.5	103.5	11.8
69	Metronidazole	2–50	1.0	92.9	9.4	97.3	5.2	99.5	13.0
70	Oxfendazole	10–250	5.0	106.7	11.8	104.5	4.8	106.6	6.7
71	Febantel	10–250	5.0	83.2	9.6	79.6	14.7	86.0	13.3
72	Fenbendazole	20–500	10.0	101.7	7.6	108.9	11.1	106.9	10.5
73	Ampicillin	10–250	5.0	112.0	8.0	111.4	6.5	103.6	6.1
74	Oxacillin	10–250	5.0	96.2	10.9	90.5	11.2	97.8	7.2
75	cloxacillin	10–250	5.0	112.1	8.5	106.1	6.4	117.2	4.6
76	Dicloxacillin	20–500	10.0	97.2	12.8	93.2	10.7	92.0	7.9
77	Penicillin G	2–50	1.0	92.8	5.3	93.3	6.6	91.3	5.7
78	Cephapirin	10–250	5.0	100.0	10.7	110.6	9.2	107.7	7.9
79	Cefpirome	2–50	1.0	97.2	8.7	87.1	14.0	91.1	10.3
80	Ceftiofur	20–500	10.0	90.9	6.7	96.3	8.6	100.3	5.4
81	Cephalexin	4–100	2.0	101.6	13.1	103.0	12.7	107.9	8.8
82	19-Nortestosterone	2–50	1.0	100.0	5.4	95.6	10.9	106.0	7.6
83	Medroxyprogesterone Acetate	2–50	1.0	79.9	10.6	82.7	12.5	85.9	8.4
84	Testosterone	2–50	1.0	94.8	6.1	90.3	10.0	87.7	4.5
85	17-Methyltestosterone	2–50	1.0	99.7	3.7	104.9	6.1	89.7	5.8
86	Chlorpromazine	1–25	0.5	84.7	3.2	80.7	5.0	87.8	4.7
87	Azaperone	0.4–10	0.2	91.7	9.3	94.9	14.3	86.4	12.1
88	Promethazine	2–50	1.0	89.8	6.5	89.1	6.6	92.4	4.5
89	Acetopromaizine	1–25	0.5	82.8	5.5	80.3	11.2	79.7	10.1
90	Diazepam	10–250	5.0	90.9	11.1	106.7	10.9	116.1	14.3
91	Doramectin	20–500	10.0	111.4	10.4	96.8	9.9	107.7	11.0
92	Ivermectin	20–500	10.0	90.6	7.4	106.1	11.9	107.6	8.2
93	Maduramicin ammonium	20–500	10.0	104.5	8.1	113.3	10.1	106.2	6.8
94	Salinomycin	20–500	10.0	108.8	9.3	100.7	4.5	91.8	12.5
95	Monensin	20–500	10.0	89.3	10.1	85.5	9.0	107.4	12.1
96	2-Quinoxalinecarbox	2–50	1.0	89.7	5.6	88.0	3.6	80.0	9.1
97	Carbadox	1–25	0.5	83.9	14.8	80.1	8.2	80.3	11.9
98	Olaquindox	10–250	5.0	109.3	12.3	98.4	14.2	101.4	11.5
99	Desoxycarbadox	1–25	0.5	86.3	10.1	95.1	11.5	107.8	12.2
100	levamisole	10–250	5.0	85.8	9.8	83.2	7.3	86.4	12.0
101	Carbofuran	2–50	1.0	97.9	9.4	96.8	12.9	86.9	9.5
102	Coumaphos	20–500	10.0	80.4	4.6	79.5	6.6	83.5	8.4
103	Fenthion-sulfone	20–500	10.0	90.2	4.0	97.3	7.7	90.5	9.1
104	Fenthion-sulfoxide	2–50	1.0	93.4	14.7	88.8	10.3	91.2	12.1
105	Malathion	2–50	1.0	81.2	10.9	82.9	10.8	81.6	14.4
106	Phoxim	2–50	1.0	82.8	5.0	79.2	5.3	80.6	11.2
107	Dipterex	2–50	1.0	103.9	9.5	107.2	4.0	101.6	7.6
108	Trimethoprim	2–50	1.0	118.0	7.4	113.1	12.7	116.8	11.3
109	Atropine	1–25	0.5	112.4	3.4	110.4	4.0	112.1	3.1
110	Procaine	2–50	1.0	88.1	2.2	89.3	8.2	88.9	4.0
111	Lignocaine	1–25	0.5	96.4	11.7	91.8	7.9	109.8	12.1
112	Scopolamine	1–25	0.5	82.1	10.4	91.7	5.7	87.5	6.7
113	Anisodamine	1–25	0.5	99.0	5.5	89.8	1.5	106.8	6.7
114	Sulfanilamide	20–500	10.0	112.4	11.0	111.7	10.6	106.2	7.2
115	Mabuterol(Ambuterol)	1–25	0.5	90.2	6.3	97.7	12.3	92.5	10.6
116	Cefazolin	2–50	1.0	96.5	4.7	86.7	9.9	89.3	3.4
117	Amantadine	2–50	1.0	110.8	10.4	118.1	7.5	100.6	14.8
118	Rimantadine	2–50	1.0	107.4	11.7	100.6	13.7	97.1	8.2
119	Ribavirin	4–100	2.0	82.7	4.9	84.0	5.7	79.8	2.9
120	Oseltamivir	2–50	1.0	113.4	8.0	100.5	6.7	115.5	3.3
121	4-Epi-Oxytetracycline	10–250	5.0	95.4	6.8	97.8	7.9	108.9	2.5
122	4-Epi-Chlortetracycline	10–250	5.0	105.1	4.6	117.2	7.5	104.2	5.3
123	4-Epi-Demeclocycline	10–250	5.0	101.6	2.8	114.3	4.6	103.9	7.6
124	Nequinate	20–500	10.0	80.1	10.0	85.7	11.0	81.9	4.6
125	Clopidol	10–250	5.0	110.7	13.3	109.4	14.9	105.6	9.4
126	Amprolium	10–250	5.0	101.4	12.6	101.6	7.4	114.0	9.0
127	Halofuginone hydrobromide	10–250	5.0	95.5	2.5	85.6	1.5	96.2	1.1
128	Narasin	20–500	10.0	81.1	4.3	79.6	11.6	82.7	7.8
129	Albendazole-2-aminosulfone	4–100	2.0	105.1	9.1	108.9	4.0	105.3	8.2
130	Albendazole sulfone	2–50	1.0	92.3	8.2	100.2	5.1	105.8	10.2
131	Albendazole sulfoxide	10–250	5.0	97.7	5.0	114.8	5.0	115.8	2.7
132	Albendazole	20–500	10.0	85.4	1.1	81.8	5.2	80.8	4.9
133	Diclazuril	20–500	10.0	89.3	2.5	100.4	4.1	93.4	6.3
134	Chloramphenicol	0.2–5	0.1	102.6	13.1	110.8	14.1	99.1	5.5
135	Beclomethasone	2–50	1.0	101.1	6.7	100.1	4.8	112.9	7.8
136	Cortisone acetate	1–25	0.5	114.9	6.7	94.3	5.5	91.9	2.8
137	Dexamethasone	1–25	0.5	91.9	14.1	97.6	7.6	96.9	7.1
138	Methylprednisolone	1–25	0.5	101.7	6.6	97.7	8.1	91.8	10.1
139	Cortisone	1–25	0.5	117.7	4.7	111.6	10.0	105.8	15.2
140	Meprednisone	10–250	5.0	112.8	2.0	114.9	9.1	95.7	9.2
141	Hydrocortisone	2–50	1.0	95.4	3.1	92.4	8.1	105.4	11.0
142	Fludrocortisone acetate	10–250	5.0	96.3	10.0	93.8	13.3	104.6	2.5
143	Betamethasone	1–25	0.5	111.5	2.4	96.7	8.9	105.4	6.5
144	Diethylstilbestrol	20–500	10.0	94.4	4.3	95.1	7.7	89.3	7.4
145	Estradiol	20–500	10.0	95.4	6.7	88.6	4.5	90.7	13.7
146	Hexestrol	20–500	10.0	80.9	12.9	81.8	9.7	86.8	13.3
147	Lasalocid	20–500	10.0	85.6	3.1	86.4	4.5	83.5	4.3
148	Fipronil	2–50	1.0	97.0	4.2	93.8	10.3	100.5	8.0
149	Clazuril	20–500	10.0	96.7	9.9	93.8	13.1	102.8	11.8
150	Nicarbazin	2–50	1.0	88.0	2.0	82.9	4.2	80.9	2.2
151	Fipronil sulfone	4–100	2.0	92.5	6.8	97.9	3.9	83.0	7.3
152	Fipronil sulfide	4–100	2.0	111.3	4.0	104.7	8.1	115.9	13.0
153	Fipronil desulfinyl	4–100	2.0	110.5	9.5	108.3	11.0	98.1	12.1
154	Thiamphenicol	2–50	1.0	98.8	11.0	94.4	10.9	92.9	7.1
155	Abamectin	20–500	10.0	98.3	12.1	85.5	6.5	103.5	5.1

## Results and Discussion

**Fig. 1:**
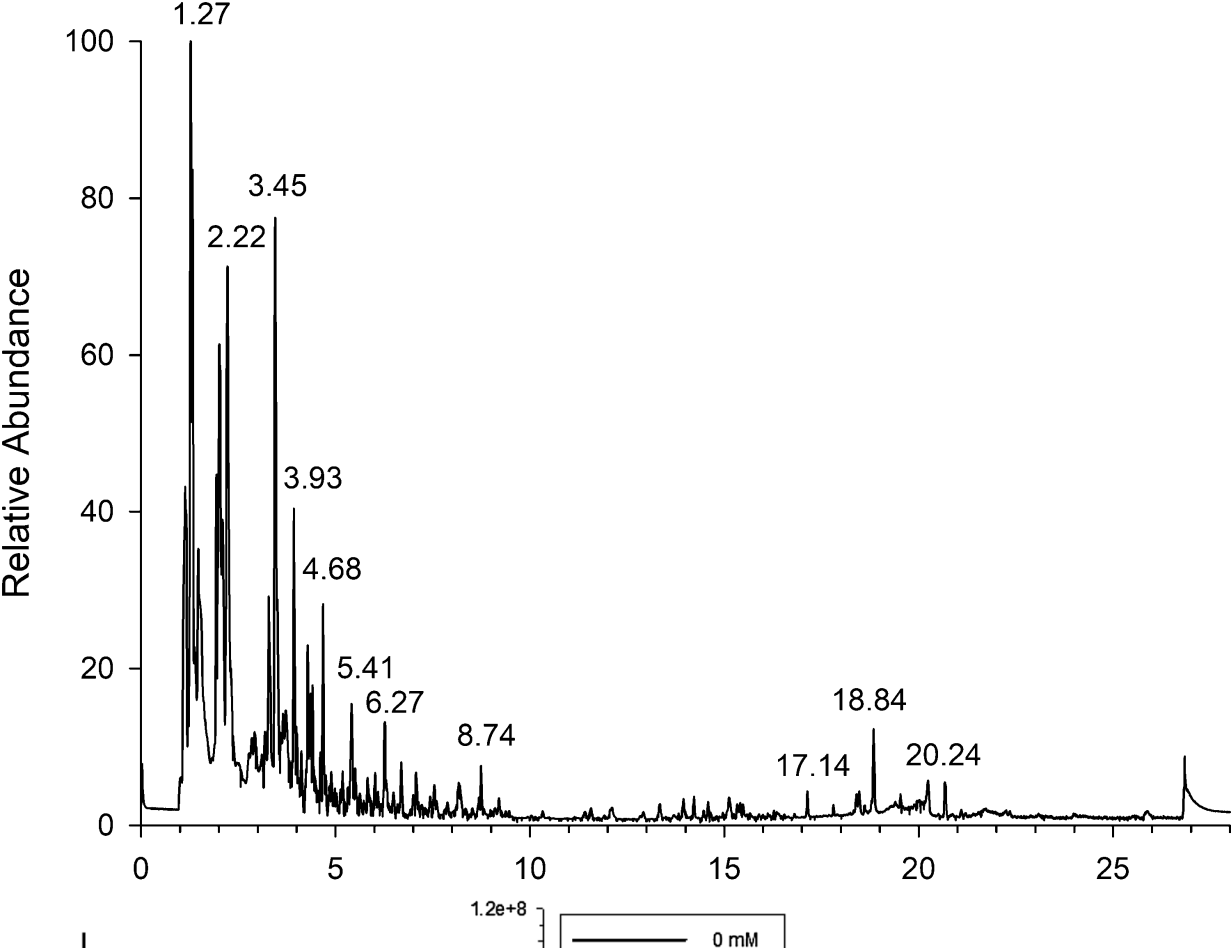
Total ion current chromatogram of 155 veterinary drugs

The 155 veterinary drugs selected in this study cover an extremely wide polarity range. Thus, chromatographic columns with good retention for compounds of different polarity degrees must be selected. First, the separation effects of 155 compounds on three chromatographic columns, including a Waters Acquity UPLC BEH C18 column, a Thermo Hypersil GOLD column, and an Agilent ZORBAX Eclipse Plus C18 column, were compared. Results showed that the separation effects and peak shapes for the Agilent ZORBAX Eclipse Plus C18 column were superior to those of the two other chromatographic columns. Furthermore, the mobile phases (0.1% FA aqueous solution and 5 mM ammonium acetate) were optimized. Organic solvents investigated were ACN and methanol, as well as mixtures of ACN solution containing 0.1% FA and methanol containing 0.1% FA solution. In the positive mode, 0.1% FA aqueous solution and ACN solution containing 0.1% FA performed best in separating the target compounds; in the negative mode, 5 mM ammonium acetate and ACN performed best. The ion flow chromatogram of 155 target compounds is shown in Fig. 1.

**Fig. 2:**
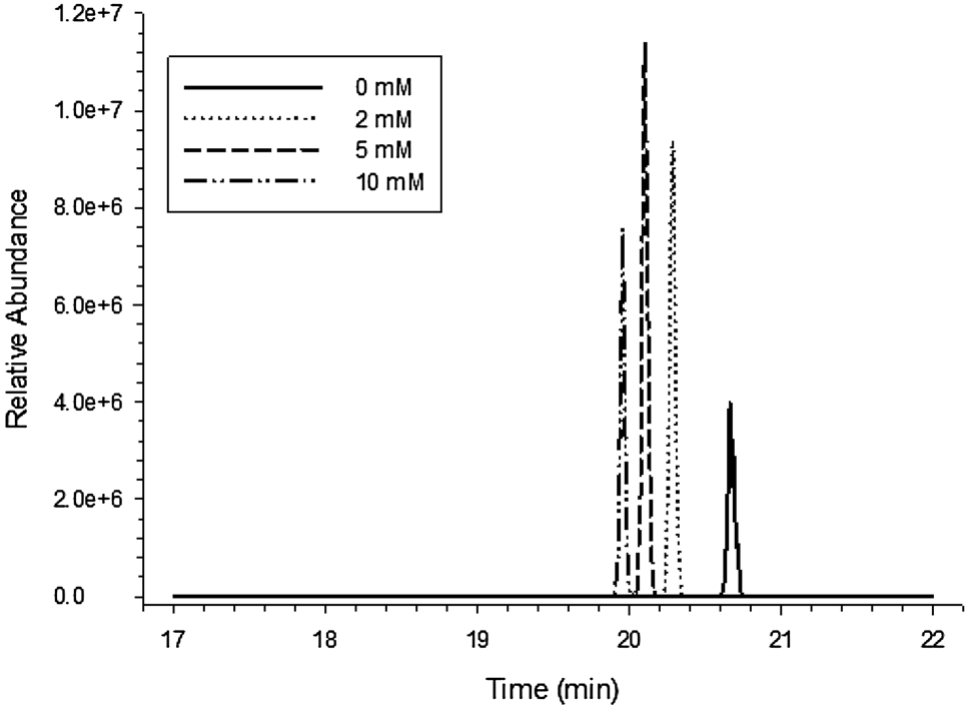
Effect of ammonium acetate concentration in mobile phase on quality spectrum response of abamectin

**Fig. 3:**
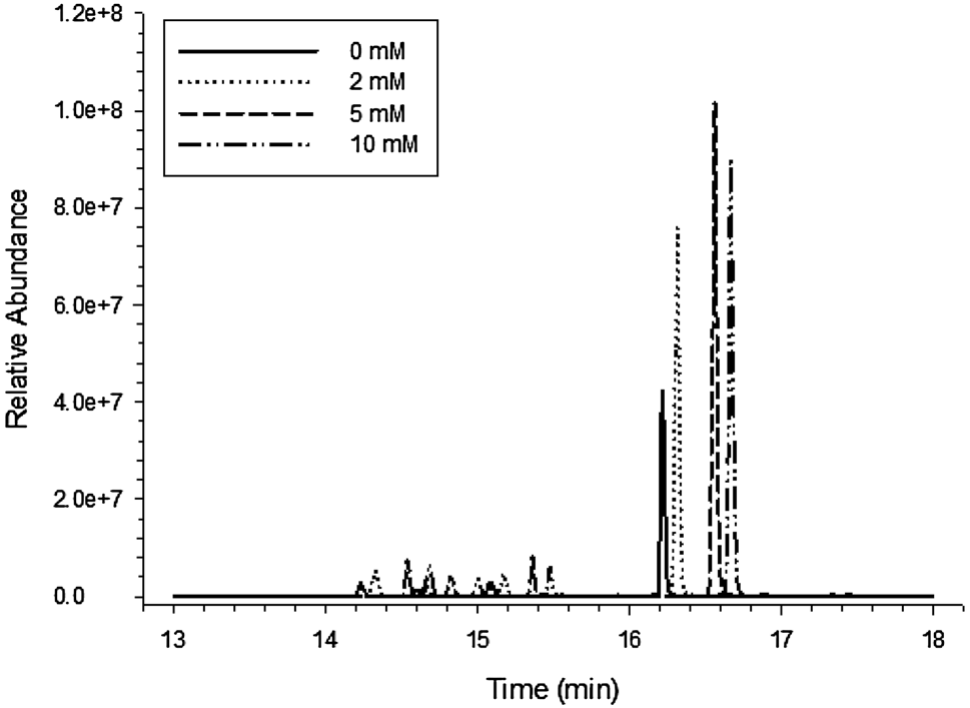
Effect of ammonium acetate concentration in mobile phase on quality spectrum response of hexestrol

Adding an appropriate amount of salt in the mobile phases can help improve the peak shape of the mass spectrum and improve the ionization efficiency of the substance to be measured. However, the ionization efficiency reduced when the concentration of salt was too high, thus resulting in the mass spectrum response of the substance to be measured. Ammonium acetate and ACN at 0, 2, 5 and 10 mM were selected as mobile phases, respectively, to investigate the chromatographic peak shape and mass spectral response intensity of each compound in the negative mode. As shown in Figs. 2 and 3, when the ammonium acetate concentration in the aqueous mobile phase was 5 mM, the responses of abamectin and hexestrol were significantly higher than those of pure water or other concentrations of ammonium acetate. Therefore, 5 mM ammonium acetate and ACN were selected as the mobile phases in the negative mode in this experiment.

The target compounds were quantitatively and qualitatively analyzed by MS. Thus, isomers were required to achieve baseline separation on the chromatographic column. Four pairs of sulfa compounds are isomers of each other (Nos. 6 and 7, 8 and 9, 13 and 16, 17 and 18), and three compounds are isomers of each other (Nos. 5, 12, and 14). Baseline separation was achieved under selected chromatographic conditions, and retention times are shown in supporting information (Table S1).

**Fig. 4:**
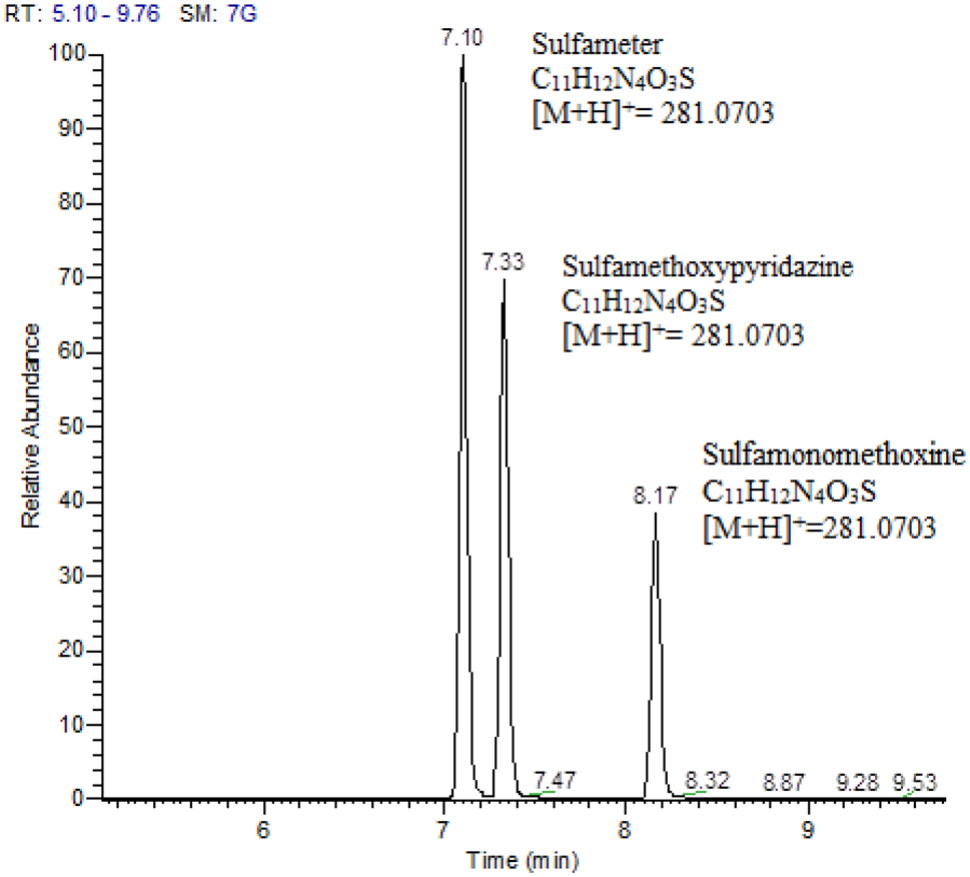
Extracted ion chromatogram of the exact mass of 281.0703 (sulfameter, sulfamethoxypyridazine, or sulfamonomethoxine)

Sulfameter, sulfamethoxypyridazine, and sulfamonomethoxine are isomers with identical plasmonucleic ratios, which must be separated by chromatographic retention time. In this study, the chromatographic conditions were optimized to achieve baseline separation. From the extracted ion chromatogram (Fig. 4), the three sulfamides were determined by peak comparison with reference standards.

The Q-Exactive Orbitrap/MS was operated in Full MS/dd MS2 scanning mode in the positive and negative modes. Targeted identification was achieved by full-scan MS; if a veterinary drug was detected, the data-dependent MS2 (dd-MS2) scan was triggered.

During the Full MS scan, the mass resolution was set at 60,000 FWHM, AGC target at 1.0E6, maximum IT 200 ms, and scan range m/z 135–1100. If the targeted compound was detected within a 5 ppm mass error window and achieved by a designated intensity threshold (i.e., a setting of 1.0E5), the precursor ions in the inclusion list were then isolated by the quadrupole and sent to the HCD collision cell for fragmentation via the C-trap. The inclusion list consists of precursor ions that are of interest for targeted identification and is provided in supporting information (Table S1). The resulting dd-MS2 product-ion spectra were generated by fragmenting the precursor ions with stepped normalized collision energy (NCE). At this stage, the mass resolution of the Orbitrap analyzer was set at 15,000 FWHM, AGC target at 1E5, maximum IT 50 ms, isolation window m/z 2.0, NCE 40% ± 50%, underfill ratio 10%, intensity threshold 8.0E4, apex trigger 1–12 s, and dynamic exclusion 12.0 s.

**Fig. 5:**
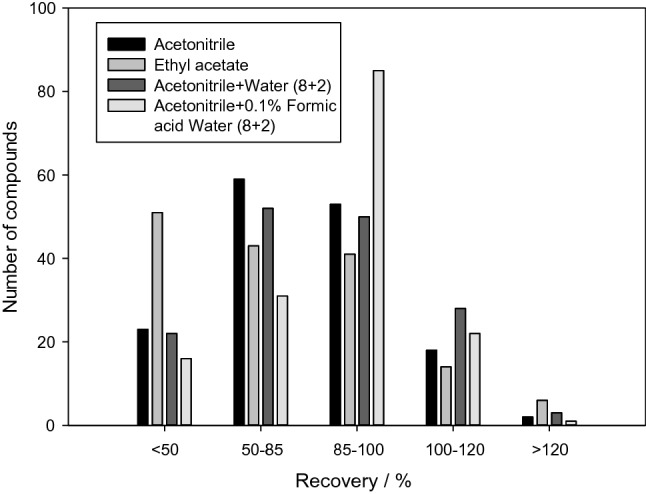
Number of compounds extracted with different extraction solvents

An appropriate extraction solvent needs to consider the properties of the solvent, veterinary drugs, and matrix. Organic solvents, such as ACN, ethyl acetate, ACN-aqueous solution, and ACN-0.2 % formic water (8 + 2) solution, were selected to extract different matrix samples. Figure 5 shows the extraction efficiencies and recovery rates of the 155 compounds in the different solvents. Results showed that ACN-0.2 % formic water (8 + 2) solution recovered more compounds in a more appropriate range (80 < R < 120) compared with the other solvents.

**Fig. 6:**
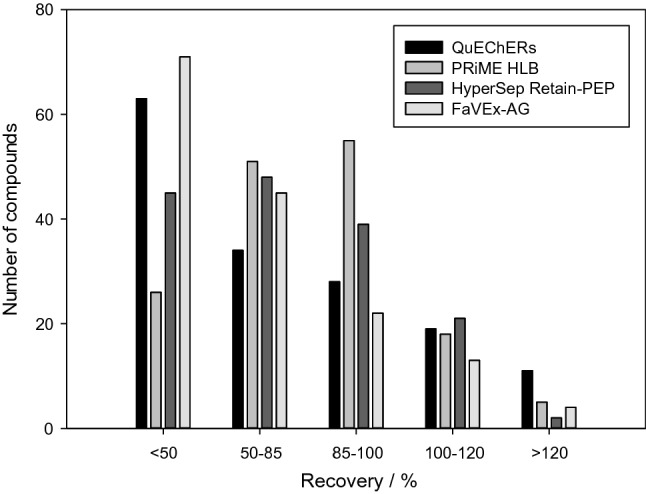
Number of compounds extracted with the different clean-up approaches evaluated

The fats and proteins in animal-derived food must be removed during purification because they increase the matrix effect, reduce the sensitivity of the method, pollute the mass spectrum, and shorten the service life of the instrument. Four purification methods, including QuEChERs purification, PRiME HLB SPE column, HyperSep Retain PEP SPE column, and Favex-AG column purification, were selected and evaluated, and the results are shown in Fig. 6. More compounds were recovered when the PRiME HLB SPE column was used for purification, where the recovery rate was within a reasonable range. Therefore, the PRiME HLB SPE column was selected as the purification method.

**Fig. 7:**
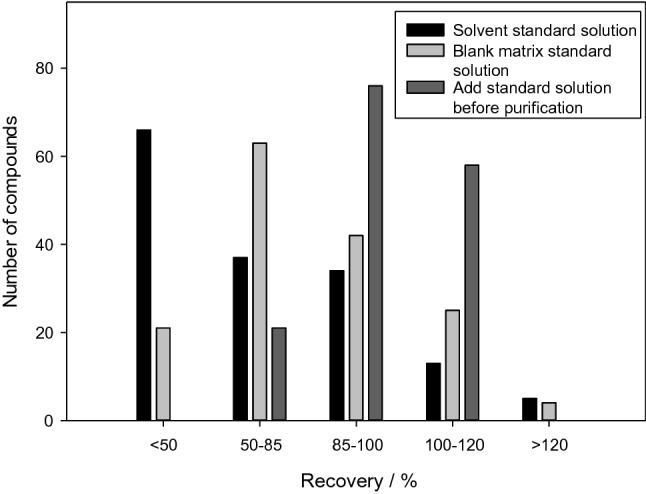
Number of compounds extracted with the different standard curve preparation methods

A calibration curve was prepared to compare the three methods, namely, using solvent standard solution, blank matrix standard solution, and add standard solution before purification, for the quantitative correction and calculation of the recovery rates of the veterinary drugs. The results are shown in Fig. 7.

When the standard curve of the solvent standard solution method was used for quantitative detection, the recovery rates of most of the 155 veterinary drugs were low because the preparation method could not correct matrix effects and because of loss of veterinary drugs during the pretreatment. For the standard curve of the blank matrix standard solution method, the recovery rate was significantly higher than that of the solvent standard solution method because it could reduce the influence of matrix effects but could not eliminate the loss of most veterinary drugs during the pretreatment. It still could not meet the need of accurate quantification. In the present study, a standard solution was added before pretreatment for the preparation of the standard curve. This method can not only reduce matrix effects but also effectively reduce wastage of the target compounds during pretreatment.

**Table 2:** Veterinary drug detection in authentic samples

Animal species (Number of samples)	Analyte	Number of positive samples	Content range µg/kg	Animal species (Number of samples)	Analyte	Number of positive samples	Content range µg/kg
Beef (40)	Hydrocortisone	34	1.36–45.4	Pork (37)	Hydrocortisone	36	1.27–13.9
	Cortisone	6	1.07–3.28		Cortisone	5	1.19–4.13
	Atropine	3	1.82–2.45		Chlortetracycline	5	5.08–11.2
	Chlortetracycline	2	5.66–339		4-Epi-Chlortetracycline	3	6.12–7.33
	Trimethoprim	2	1.12–5.08		Tilmicosin	4	1.11–11.5
	Enrofloxacin	2	9.07–13.8		Atropine	1	1.91
	Clenbuterol	1	1.05		Levamisole	1	5.90
	Florfenicol	1	51.8		Thiabendazole	1	0.571
	Ciprofloxacin	1	4.18	Mutton (36)	Hydrocortisone	32	1.22–13.5
	Thiabendazole	1	0.536		Chlortetracycline	10	5.03–103
	Doxycycline	1	171		4-Epi-Chlortetracycline	7	5.70–56.2
	Tetracycline	1	14.2		Cortisone	3	1.58–2.13
	4-Epi-Chlortetracycline	1	157		Enrofloxacin	2	3.88–4.06
	Sulfisomidine	1	21.6		Doxycycline	1	132
	Testosterone	1	1.07		Trimethoprim	1	1.79
Pork liver (36)	Chlortetracycline	11	6.21–147	Chicken (24)	Thiabendazole	2	0.635–1.14
	4-Epi-Chlortetracycline	9	8.79–103		Enrofloxacin	1	4.27
	Enrofloxacin	5	3.75–8.05		Oxytetracycline	1	11.4
	Tilmicosin	5	2.49–177		Diclazuril	1	26.0
	Olaquindox	4	1.65–19.0	Chicken liver (22)	Tilmicosin	4	1.45–179
	Doxycycline	3	8.46–29.6		Chlortetracycline	3	10.4–31.7
	Ofloxacin	1	225		4-Epi-Chlortetracycline	3	6.77–23.2
	Lincomycin	1	4.03		Doxycycline	2	11.7–18.1
	Trimethoprim	1	1.2		Amantadine	1	29.7
	Lignocaine	1	0.505		Diclazuril	1	320

The method was applied to 195 meat and viscera samples, in cloud pork, beef, mutton, chicken, pork liver, and chicken liver. The sample information is provided in supporting information (Table S2). The results are shown in Table 2. Fourteen classes of veterinary drugs, such as quinolones, agonists, tetracyclines, sulfamides, and glucocorticoids, were detected in the samples, among which hydrocortisone and cortisone were endogenous glucocorticoids. Meanwhile, 22 classes of veterinary drugs were detected in 70 samples, with a detection rate of 35.9 %. Aureomycin was detected in 31 samples, which was the most veterinary drug detected, with contents ranging from 5.03 µg/kg to 147 µg/kg. Its metabolite chlorquatrimycin was detected in 21 samples, ranging from 5.70 µg/kg to 157 µg/kg. Timicoxacin was detected in 13 samples, mainly concentrated in pig liver and chicken liver, ranging from 1.11 µg/kg to 179 µg/kg. The detection rates of enoxacin and doxycycline were also relatively high.

Therefore, the detection rates of veterinary drug residues in animal source foods rapidly screened by high-resolution MR are higher than those for other methods because of its higher sensitivity than HPLC-MS/MS and lower LOD than conventional detection standards. Moreover, it covered 155 types of veterinary drugs. Thus, such a detection range cannot be achieved by conventional detection methods. In addition, a large number of veterinary drugs were detected in 195 authentic samples, but the values of the results were less than the LOQ. These data may provide insights into breeding animals for human consumption.

## Conclusions

UPLC-Q-Exactive Orbitrap/MS was used to establish a fast method for the qualitative and quantitative analysis of 155 veterinary drug residues of different classes in animal source foods. With this method, high-resolution MS can ensure the elimination of interference in complex matrix backgrounds and significantly improve the accuracy of qualitative and quantitative analyses, and data-dependent, full-scan, secondary-ion, MS further improves the accuracy of qualitative results. This method can increase the detection flux, reduce the detection cost, shorten the detection cycle, and provide technical support for the rapid response to food safety problems and the detection of potential food safety risks.

## Supplementary Information

Below is the link to the electronic supplementary material.

Electronic supplementary material 1 (DOCX 3093 kb)
