# Supplementary material for: Screening and Analysis of Multiclass Veterinary Drug Residues in Animal Source Foods using UPLC-Q-Exactive Orbitrap/MS
Source: Bull Environ Contam Toxicol. 2021 Jun 7;107(2):228–38. doi: 10.1007/s00128-021-03273-w (PMC8346397; doi:10.1007/s00128-021-03273-w)
Supplement: Supplementary file 1 — Electronic supplementary material 1 (DOCX 3093 kb) [file 128_2021_3273_MOESM1_ESM.docx]

**Supporting information**

Table S1 List of 155 veterinary drugs targeted in the study

| Number | Analytes | Drug Class | CAS Number | Elemental composition | Ionization | Exact mass | Retention time (min) |
| --- | --- | --- | --- | --- | --- | --- | --- |
| 1 | Sulfaphenazole | Sulfonamide | 526-08-9 | C_15_H_14_N_4_O_2_S | [M+H]^+^ | 315.0910 | 11.9 |
| 2 | Sulfabenzamide | Sulfonamide | 127-71-9 | C_13_H_12_N_2_O_3_S | [M+H]^+^ | 277.0641 | 11.2 |
| 3 | Sulfapyridine | Sulfonamide | 144-83-2 | C_11_H_11_N_3_O_2_S | [M+H]^+^ | 250.0645 | 5.7 |
| 4 | Sulfacetamide | Sulfonamide | 144-80-9 | C_8_H_10_N_2_O_3_S | [M+H]^+^ | 215.0485 | 5.0 |
| 5 | Sulfameter | Sulfonamide | 651-06-9 | C_11_H_12_N_4_O_3_S | [M+H]^+^ | 281.0703 | 7.4 |
| 6 | Sulfamoxole | Sulfonamide | 729-99-7 | C_11_H_13_N_3_O_3_S | [M+H]^+^ | 268.0750 | 6.4 |
| 7 | Sulfisoxazole | Sulfonamide | 127-69-5 | C_11_H_13_N_3_O_3_S | [M+H]^+^ | 268.0750 | 10.1 |
| 8 | Sulfamethazine | Sulfonamide | 57-68-1 | C_12_H_14_N_4_O_2_S | [M+H]^+^ | 279.0910 | 4.6 |
| 9 | Sulfisomidine | Sulfonamide | 515-64-0 | C_12_H_14_N_4_O_2_S | [M+H]^+^ | 279.0910 | 6.8 |
| 10 | Sulfamethoxazole | Sulfonamide | 723-46-6 | C_10_H_11_N_3_O_3_S | [M+H]^+^ | 254.0594 | 9.5 |
| 11 | Sulfamerazine | Sulfonamide | 127-79-7 | C_11_H_12_N_4_O_2_S | [M+H]^+^ | 265.0754 | 6.1 |
| 12 | Sulfamethoxypyridazine | Sulfonamide | 80-35-3 | C_11_H_12_N_4_O_3_S | [M+H]^+^ | 281.0703 | 7.2 |
| 13 | Sulfadimethoxypyrimidine | Sulfonamide | 155-91-9 | C_12_H_14_N_4_O_4_S | [M+H]^+^ | 311.0809 | 11.4 |
| 14 | Sulfamonnomethoxine | Sulfonamide | 1220-83-3 | C_11_H_12_N_4_O_3_S | [M+H]^+^ | 281.0703 | 8.2 |
| 15 | Sulfaquinoxaline | Sulfonamide | 59-40-5 | C_14_H_12_N_4_O_2_S | [M+H]^+^ | 301.0754 | 11.5 |
| 16 | Sulfadimoxine | Sulfonamide | 2447-57-6 | C_12_H_14_N_4_O_4_S | [M+H]^+^ | 311.0809 | 9.1 |
| 17 | Sulfaclozine | Sulfonamide | 102-65-8 | C_10_H_9_ClN_4_O_2_S | [M+H]^+^ | 285.0208 | 11.2 |
| 18 | Sulfachloropyridazine | Sulfonamide | 80-32-0 | C_10_H_9_ClN_4_O_2_S | [M+H]^+^ | 285.0208 | 8.8 |
| 19 | Sulfaguanidine | Sulfonamide | 59-40-5 | C_7_H_10_N_4_O_2_S | [M+H]^+^ | 215.0597 | 3.1 |
| 20 | Sulfadiazine | Sulfonamide | 68-53-9 | C_10_H_10_N_4_O_2_S | [M+H]^+^ | 251.0597 | 5.3 |
| 21 | Sulfathiazole | Sulfonamide | 72-14-0 | C_9_H_9_N_3_O_2_S_2_ | [M+H]^+^ | 256.0209 | 5.6 |
| 22 | Sulfamethizole | Sulfonamide | 144-82-1 | C_9_H_10_N_4_O_2_S_2_ | [M+H]^+^ | 271.0318 | 7.1 |
| 23 | Orbifloxacin | Fluoroquinolone | 113617-63-3 | C_19_H_20_F_3_N_3_O_3_ | [M+H]^+^ | 396.1530 | 6.7 |
| 24 | Danofloxacin | Fluoroquinolone | 112398-08-0 | C_19_H_20_FN_3_O_3_ | [M+H]^+^ | 358.1562 | 6.2 |
| 25 | Enrofloxacin | Fluoroquinolone | 93106-60-6 | C_19_H_22_FN_3_O_3_ | [M+H]^+^ | 360.1718 | 6.5 |
| 26 | Flumequine | Fluoroquinolone | 42835-25-6 | C_14_H_12_FNO_3_ | [M+H]^+^ | 262.0874 | 14.0 |
| 27 | Fleroxacin | Fluoroquinolone | 79660-72-3 | C_17_H_18_F_3_N_3_O_3_ | [M+H]^+^ | 370.1373 | 5.8 |
| 28 | Ciprofloxacin | Fluoroquinolone | 85721-33-1 | C_17_H_18_FN_3_O_3_ | [M+H]^+^ | 332.1405 | 5.9 |
| 29 | Lomefloxacin | Fluoroquinolone | 98079-51-7 | C_17_H_19_F_2_N_3_O_3_ | [M+H]^+^ | 352.1467 | 6.2 |
| 30 | Nalidixic acid | Fluoroquinolone | 389-08-2 | C_12_H_12_N_2_O_3_ | [M+H]^+^ | 233.0921 | 13.3 |
| 31 | Norfloxacin | Fluoroquinolone | 70458-96-7 | C_16_H_18_FN_3_O_3_ | [M+H]^+^ | 320.1405 | 5.7 |
| 32 | Pefloxacin | Fluoroquinolone | 70458-92-3 | C_17_H_20_FN_3_O_3_ | [M+H]^+^ | 334.1562 | 5.9 |
| 33 | Sarafloxacin | Fluoroquinolone | 98105-99-8 | C_20_H_17_F_2_N_3_O_3_ | [M+H]^+^ | 386.1311 | 7.5 |
| 34 | Difloxacin | Fluoroquinolone | 98106-17-3 | C_21_H_19_F_2_N_3_O_3_ | [M+H]^+^ | 400.1467 | 7.6 |
| 35 | Sparfloxacin | Fluoroquinolone | 110871-86-8 | C_19_H_22_F_2_N_4_O_3_ | [M+H]^+^ | 393.1733 | 7.6 |
| 36 | Enoxacin | Fluoroquinolone | 74011-58-8 | C_15_H_17_FN_4_O_3_ | [M+H]^+^ | 321.1358 | 5.6 |
| 37 | Ofloxacin | Fluoroquinolone | 82419-36-1 | C_18_H_20_FN_3_O_4_ | [M+H]^+^ | 362.1511 | 5.8 |
| 38 | Clenbuterol | β-Agonist | 37148-27-9 | C_12_H_18_C_l2_N_2_O | [M+H]^+^ | 277.0869 | 7.1 |
| 39 | Ractopamine | β-Agonist | 97825-25-7 | C_18_H_23_NO_4_ | [M+H]^+^ | 302.1751 | 6.2 |
| 40 | Clorprenaline | β-Agonist | 3811-25-4 | C_11_H_16_ClNO | [M+H]^+^ | 214.0993 | 6.1 |
| 41 | Penbutolol | β-Agonist | 36507-48-9 | C_18_H_29_NO_2_ | [M+H]^+^ | 292.2271 | 14.0 |
| 42 | Metaproterenol | β-Agonist | 586-06-1 | C_11_H_17_NO_3_ | [M+H]^+^ | 212.1281 | 3.6 |
| 43 | Formoterol | β-Agonist | 73573-87-2 | C_19_H_24_N_2_O_4_ | [M+H]^+^ | 345.1809 | 7.3 |
| 44 | Fenoterol | β-Agonist | 13392-18-2 | C_17_H_21_NO_4_ | [M+H]^+^ | 304.1543 | 5.1 |
| 45 | Cimbuterol | β-Agonist | 54239-39-3 | C_13_H_19_N_3_O | [M+H]^+^ | 234.1601 | 4.6 |
| 46 | Bambuterol | β-Agonist | 81732-65-2 | C_18_H_29_N_3_O_5_ | [M+H]^+^ | 368.2180 | 8.2 |
| 47 | Phenylethanolamine A | β-Agonist | 1346746-81-3 | C_19_H_24_N_2_O_4_ | [M+H]^+^ | 345.1809 | 11.6 |
| 48 | Thiabendazole | β-Agonist | 148-79-8 | C_10_H_7_N_3_S | [M+H]^+^ | 202.0433 | 5.1 |
| 49 | Salbutamol | β-Agonist | 18559-94-9 | C_13_H_21_NO_3_ | [M+H]^+^ | 240.1594 | 3.9 |
| 50 | Terbutaline | β-Agonist | 23031-25-6 | C_12_H_19_NO_3_ | [M+H]^+^ | 226.1438 | 3.9 |
| 51 | Tulobuterol | β-Agonist | 41570-61-0 | C_12_H_18_ClNO | [M+H]^+^ | 228.1150 | 7.0 |
| 52 | Cimaterol | β-Agonist | 54239-37-1 | C_12_H_17_N_3_O | [M+H]^+^ | 220.1444 | 4.1 |
| 53 | Florfenicol | β-Agonist | 73231-34-2 | C_12_H_14_Cl_2_FNO_4_S | [M+H]^+^ | 358.0077 | 6.6 |
| 54 | Chlorotetracycline | Tetracycline | 57-62-5 | C_22_H_23_ClN_2_O_8_ | [M+H]^+^ | 479.1216 | 8.2 |
| 55 | Doxycycline | Tetracycline | 564-25-0 | C_22_H_24_N_2_O_8_ | [M+H]^+^ | 445.1605 | 9.1 |
| 56 | Tetracycline | Tetracycline | 60-54-8 | C_22_H_24_N_2_O_8_ | [M+H]^+^ | 445.1605 | 6.4 |
| 57 | Oxytetracycline | Tetracycline | 6153-64-6 | C_22_H_24_N_2_O_9_ | [M+H]^+^ | 461.1555 | 7.0 |
| 58 | Erythromycin | Macrolide | 59319-72-1 | C_37_H_67_NO_13_ | [M+H]^+^ | 734.4685 | 10.3 |
| 59 | Kitasamycin | Macrolide | 1392-21-8 | C_40_H_67_NO_14_ | [M+H]^+^ | 786.4634 | 13.8 |
| 60 | Lincomycin | Macrolide | 154-21-2 | C_18_H_34_N_2_O_6_S | [M+H]^+^ | 407.2210 | 4.7 |
| 61 | [Tylosin](javascript:showMsgDetail('ProductSynonyms.aspx?CBNumber=CB7708088&postData3=CN&SYMBOL_Type=A');) | Macrolide | 1401-69-0 | C_46_H_77_NO_17_ | [M+H]^+^ | 916.5264 | 11.2 |
| 62 | Tilmicosin | Macrolide | 108050-54-0 | C_46_H_80_N_2_O_13_ | [M+H]^+^ | 869.5733 | 9.2 |
| 63 | Tylosin 3-acetate 4B-(3-methylbutanoate) (2R,3R)-2,3-dihydroxybutanedioate | Macrolide | 63428-13-7 | C_57_H_93_NO_25_ | [M+H]^+^ | 1042.5945 | 15.2 |
| 64 | Oleandomycin | Macrolide | 7060-74-4 | C_35_H_61_NO_12_ | [M+H]^+^ | 688.4267 | 13.6, |
| 65 | Ronidazole | Nitroimidazoles | 7681-76-7 | C_6_H_8_N_4_O_5_ | [M+H]^+^ | 201.0618 | 5.2 |
| 66 | Dimetridazole | Nitroimidazoles | 551-92-8 | C_5_H_7_N_3_O_2_ | [M+H]^+^ | 142.0611 | 5.1 |
| 67 | Metronidazole-hydroxy | Nitroimidazoles | 4812-40-2 | C_6_H_9_N_3_O_4_ | [M+H]^+^ | 188.0666 | 4.1 |
| 68 | Dimetridazolr-hydroxy | Nitroimidazoles | 936-05-0 | C_5_H_7_N_3_O_3_ | [M+H]^+^ | 158.0560 | 4.6 |
| 69 | Metronidazole | Nitroimidazoles | 443-48-1 | C_6_H_9_N_3_O_3_ | [M+H]^+^ | 172.0717 | 4.5 |
| 70 | Oxfendazole | Antinematodal drug | 53716-50-0 | C_15_H_13_N_3_O_3_S | [M+H]^+^ | 316.0750 | 9.0 |
| 71 | Febantel | Antinematodal drug | 58306-30-2 | C_20_H_22_N_4_O_6_S | [M+H]^+^ | 447.1333 | 17.1 |
| 72 | Fenbendazole | Antinematodal drug | 43210-67-9 | C_15_H_13_N_3_O_2_S | [M+H]^+^ | 300.0801 | 14.8 |
| 73 | Ampicillin | β-Lactam | 7177-48-2 | C_16_H_19_N_3_O_4_S | [M+H]^+^ | 350.1169 | 5.3 |
| 74 | Oxacillin | β-Lactam | 66-79-5 | C_19_H_19_N_3_O_5_S | [M+H]^+^ | 402.1118 | 14.8 |
| 75 | Cloxacillin | β-Lactam | 61-72-3 | C_19_H_18_ClN_3_O_5_S | [M+H]^+^ | 436.0729 | 15.2 |
| 76 | Dicloxacillin | β-Lactam | 3116-76-5 | C_19_H_17_Cl_2_N_3_O_5_S | [M+H]^+^ | 470.0339 | 15.9 |
| 77 | Penicillin G | β-Lactam | 113-98-4 | C_16_H_18_N_2_O_4_S | [M+H]^+^ | 335.1060 | 5.8 |
| 78 | Cephapirin | Cephalosporins | 24356-60-3 | C_17_H_17_N_3_O_6_S_2_ | [M+H]^+^ | 424.0632 | 4.1 |
| 79 | Cefpirome | Cephalosporins | 84957-30-2 | C_23_H_24_N_6_O_5_S_2_ | [M+H]^+^ | 529.1322 | 4.6 |
| 80 | Ceftiofur | Cephalosporins | 80370-57-6 | C_19_H_17_N_5_O_7_S_3_ | [M+H]^+^ | 524.0363 | 10.2 |
| 81 | Cephalexin | Cephalosporins | 15686-71-2 | C_16_H_17_N_3_O_4_S | [M+H]^+^ | 348.1013 | 5.3 |
| 82 | 19-Nortestosterone | Sex hormone | 434-22-0 | C_18_H_26_O_2_ | [M+H]^+^ | 275.2006 | 15.1 |
| 83 | Medroxyprogesterone Acetate | Sex hormone | 71-58-9 | C_24_H_34_O_4_ | [M+H]^+^ | 387.2530 | 17.8 |
| 84 | Testosterone | Sex hormone | 58-22-0 | C_19_H_28_O_2_ | [M+H]^+^ | 289.2162 | 15.6 |
| 85 | 17-Methyltestosterone | Sex hormone | 58-18-4 | C_20_H_30_O_2_ | [M-HCOOH+H]^+^ | 303.2319 | 16.1 |
| 86 | Chlorpromazine | Sedative | 50-53-3 | C_17_H_19_ClN_2_S | [M+H]^+^ | 319.1030 | 14.3 |
| 87 | Azaperone | Sedative | 1649-18-9 | C_19_H_22_FN_3_O | [M+H]^+^ | 328.1820 | 6.4 |
| 88 | Promethazine | Sedative | 60-87-7 | C_17_H_20_N_2_S | [M+H]^+^ | 285.1420 | 12.3 |
| 89 | Acetopromaizine | Sedative | 61-00-7 | C_19_H_22_N_2_OS | [M+H]^+^ | 327.1526 | 12.1 |
| 90 | Diazepam | Sedative | 439-14-5 | C_16_H_13_ClN_2_O | [M+H]^+^ | 285.0789 | 15.9 |
| 91 | Doramectin | Coccidiostat | 117704-25-3 | C_50_H_74_O_14_ | [M+H]^+^ | 899.5151 | 21.4 |
| 92 | Ivermectin | Coccidiostat | 70288-86-7 | C_48_H_74_O_14_ | [M+Na]^+^ | 897.4971 | 9.2 |
| 93 | [Maduramicin ammonium](javascript:showMsgDetail('ProductSynonyms.aspx?CBNumber=CB9703623&postData3=CN&SYMBOL_Type=A');) | Coccidiostat | 84878-61-5 | C_47_H_80_O_17_H_3_N | [M+H]^+^ | 934.5734 | 20.4 |
| 94 | Salinomycin | Coccidiostat | 53003-10-4 | C_42_H_69_NaO_11_ | [M+H]^+^ | 773.4810 | 20.0 |
| 95 | Monensin | Coccidiostat | 22373-78-0 | C_36_H_62_O_11_ | [M+Na]^+^ | 693.4184 | 25.6 |
| 96 | 2-Quinoxalinecarbox | Synthetic antibacterials | 879-65-2 | C_9_H_6_N_2_O_2_ | [M+H]^+^ | 175.0502 | 6.3 |
| 97 | Carbadox | Synthetic antibacterials | 6804-7-5 | C_11_H_10_N_4_O_4_ | [M+H]^+^ | 263.0775 | 5.9 |
| 98 | Olaquindox | Synthetic antibacterials | 23696-28-8 | C_10_H_8_N_2_O_2_ | [M+H]^+^ | 189.0659 | 6.6 |
| 99 | Desoxycarbadox | Synthetic antibacterials | 55456-55-8 | C_11_H_10_N_4_O_2_ | [M+H]^+^ | 231.0877 | 9.2 |
| 100 | Levamisole | Nitroimidazoles | 14769-73-4 | C_11_H_12_N_2_S | [M+H]^+^ | 205.0794 | 5.0 |
| 101 | Carbofuran | Helminthic | 1563-66-2 | C_12_H_15_NO_3_ | [M+H]^+^ | 222.1125 | 14.2 |
| 102 | Coumaphos | Helminthic | 56-72-4 | C_14_H_16_ClO_5_PS | [M+H]^+^ | 363.0217 | 18.0 |
| 103 | Fenthion-sulfone | Helminthic | 3761-42-0 | C_10_H_15_O_5_PS_2_ | [M+H]^+^ | 311.0171 | 15.9 |
| 104 | Fenthion-sulfoxide | Helminthic | 3761-41-9 | C_10_H_15_O_4_PS_2_ | [M+H]^+^ | 295.0222 | 14.2 |
| 105 | Malathion | Helminthic | 121-75-5 | C_10_H_19_O_6_PS_2_ | [M+H]^+^ | 331.0433 | 17.2 |
| 106 | Phoxim | Helminthic | 14816-18-3 | C_12_H_15_N_2_O_3_PS | [M+H]^+^ | 299.0614 | 21.2 |
| 107 | Dipterex | Helminthic | 52-68-6 | C_4_H_8_C_l3_O_4_P | [M+H]^+^ | 256.9299 | 7.2 |
| 108 | Trimethoprim | Sulfonamide | 738-70-5 | C_14_H_18_N_4_O_3_ | [M+H]^+^ | 291.1452 | 5.5 |
| 109 | Atropine | Water-retaining drug | 52-55-8 | C_17_H_23_NO_3_ | [M+H]^+^ | 290.1751 | 6.0 |
| 110 | Procaine | Water-retaining drug | 59-46-1 | C_13_H_20_N_2_O_2_ | [M+H]^+^ | 237.1598 | 4.5 |
| 111 | Lignocaine | Water-retaining drug | 137-58-6 | C_14_H_22_N_2_O | [M+H]^+^ | 235.1805 | 6.1 |
| 112 | Scopolamine | Water-retaining drug | 6533-68-2 | C_17_H_21_NO_4_ | [M+H]^+^ | 304.1543 | 5.1 |
| 113 | Anisodamine | Water-retaining drug | 55869-99-3 | C_17_H_23_NO_4_ | [M+H]^+^ | 306.1700 | 4.8 |
| 114 | Sulfanilamide | Sulfonamide | 63-74-1 | C_6_H_8_N_2_O_2_S | [M+H]^+^ | 173.0379 | 3.6 |
| 115 | Mabuterol(Ambuterol) | β-Agonist | 56341-08-3 | C_13_H_18_ClF_3_N_2_O | [M+H]^+^ | 311.1133 | 8.7 |
| 116 | Cefazolin | Cephalosporins | 25953-19-9 | C_14_H_14_N_8_O_4_S_3_ | [M+H]^+^ | 455.0373 | 6.8 |
| 117 | Amantadine | Antiviral | 768-94-5 | C_10_H_17_N | [M+H]^+^ | 152.1434 | 5.6 |
| 118 | Rimantadine | Antiviral | 1501-84-4 | C_12_H_21_N | [M+H]^+^ | 180.1747 | 8.8 |
| 119 | Ribavirin | Antiviral | 36791-04-5 | C_8_H_12_N_4_O_5_ | [M+H]^+^ | 245.0881 | 2.0 |
| 120 | Oseltamivir | Antiviral | 196618-13-0 | C_16_H_28_N_2_O_4_ | [M+H]^+^ | 313.2122 | 9.0 |
| 121 | 4-Epi-Oxytetracycline | Tetracycline | 14206-58-7 | C_22_H_24_N_2_O_9_ | [M+H]^+^ | 461.1555 | 4.5 |
| 122 | 4-Epi-Chlortetracycline | Tetracycline | 14297-93-9 | C_22_H_23_ClN_2_O_8_ | [M+H]^+^ | 479.1216 | 6.2 |
| 123 | 4-Epi-Demeclocycline | Tetracycline | 127-33-3 | C_21_H_21_ClN_2_O_8_ | [M+H]^+^ | 465.1059 | 7.4 |
| 124 | Nequinate | Coccidiostat | 13997-19-8 | C_22_H_23_NO_4_ | [M+H]^+^ | 366.1700 | 16.6 |
| 125 | Clopidol | Coccidiostat | 2971-90-6 | C_7_H_7_Cl_2_NO | [M+H]^+^ | 191.9978 | 4.8 |
| 126 | Amprolium | Coccidiostat | 137-88-2 | C_14_H_18_N_4_ | [M+H]^+^ | 243.1604 | 2.0 |
| 127 | Halofuginone hydrobromide | Coccidiostat | 64924-67-0 | C_16_H_17_BrClN_3_O_3_ | [M+H]^+^ | 414.0215 | 9.0 |
| 128 | Narasin | Coccidiostat | 55134-13-9 | C_43_H_72_O_11_ | [M+Na]^+^ | 787.4967 | 20.7 |
| 129 | Albendazole-2-aminosulfone | Nitroimidazoles | 80983-34-2 | C_10_H_13_N_3_O_2_S | [M+H]^+^ | 240.0801 | 5.1 |
| 130 | Albendazole sulfone | Nitroimidazoles | 75184-71-3 | C_12_H_15_N_3_O_4_S | [M+H]^+^ | 298.0856 | 9.2 |
| 131 | Albendazole sulfoxide | Nitroimidazoles | 54029-12-8 | C_12_H_15_N_3_O_3_S | [M+H]^+^ | 282.0907 | 6.5 |
| 132 | Albendazole | Nitroimidazoles | 54965-21-8 | C_12_H_15_N_3_O_2_S | [M+H]^+^ | 266.0958 | 12.0 |
| 133 | Diclazuril | Coccidiostat | 101831-37-2 | C_17_H_9_Cl_3_N_4_O_2_ | [M-H]^-^ | 404.9716 | 17.1 |
| 134 | Chloramphenicol | β-Lactams | 56-75-7 | C_11_H_12_Cl_2_N_2_O_5_ | [M-H]^-^ | 321.0051 | 10.2 |
| 135 | Beclomethasone | Glucocorticoid | 4419-39-0 | C_22_H_29_ClO_5_ | [M-H]^-^ | 407.1631 | 14.2 |
| 136 | Cortisone acetate | Glucocorticoid | 50-04-4 | C_23_H_30_O_6_ | [M-H]^-^ | 401.1970 | 15.4 |
| 137 | Dexamethasone | Glucocorticoid | 50-02-2 | C_22_H_29_FO_5_ | [M+HCOO]^-^ | 437.1981 | 13.8 |
| 138 | Methylprednisolone | Glucocorticoid | 83-43-2 | C_22_H_30_O_5_ | [M+HCOO]^-^ | 419.2075 | 13.0 |
| 139 | Cortisone | Glucocorticoid | 53-06-5 | C_21_H_28_O_5_ | [M+HCOO]^-^ | 405.1919 | 11.7 |
| 140 | Meprednisone | Glucocorticoid | 1247-42-3 | C_22_H_28_O_5_ | [M-H]^-^ | 371.1864 | 15.4 |
| 141 | Hydrocortisone | Glucocorticoid | 50-23-7 | C_21_H_30_O_5_ | [M+HCOO]^-^ | 407.2075 | 11.4 |
| 142 | Fludrocortisone acetate | Glucocorticoid | 514-36-3 | C_23_H_31_FO_6_ | [M-H]^-^ | 421.2032 | 15.2 |
| 143 | Betamethasone | Glucocorticoid | 378-44-9 | C_22_H_29_FO_5_ | [M+HCOO]^-^ | 437.1981 | 13.6 |
| 144 | Diethylstilbestrol | Sex hormone | 6898-97-1 | C_18_H_20_O_2_ | [M+HCOO]^-^ | 313.1445 | 17.3 |
| 145 | Estradiol | Sex hormone | 50-28-2 | C_18_H_24_O_2_ | [M+HCOO]^-^ | 317.1758 | 15.2 |
| 146 | Hexestrol | Sex hormone | 84-16-2 | C_18_H_22_O_2_ | [M-H]^-^ | 269.1547 | 17.8 |
| 147 | Lasalocid | Coccidiostat | 25999-20-6 | C_34_H_53_O_8_Na | [M-H]^-^ | 589.3746 | 14.6 |
| 148 | Fipronil | Helminthic | 120068-37-3 | C_12_H_4_Cl_2_F_6_N_4_OS | [M-H]^-^ | 434.9314 | 17.5 |
| 149 | Clazuril | Coccidiostat | 101831-36-1 | C_17_H_10_Cl_2_N_4_O_2_ | [M-H]^-^ | 371.0108 | 12.7 |
| 150 | Nicarbazin | Coccidiostat | 330-95-0 | C_13_H_10_N_4_O_5_ | [M-H]^-^ | 301.0578 | 16.6 |
| 151 | Fipronil sulfone | Helminthic | 120068-36-2 | C_12_H_4_Cl_2_F_6_N_4_O_2_S | [M-H]^-^ | 450.9263 | 17.9 |
| 152 | Fipronil sulfide | Helminthic | 120067-83-6 | C_12_H_4_Cl_2_F_6_N_4_S | [M-H]^-^ | 418.9365 | 17.9 |
| 153 | Fipronil desulfinyl | Helminthic | 205650-65-3 | C_12_H_4_Cl_2_F_6_N_4_ | [M-H]^-^ | 386.9644 | 17.7 |
| 154 | Thiamphenicol | β-Lactams | 15318-45-3 | C_12_H_15_Cl_2_NO_5_S | [M-H]^-^ | 353.9975 | 6.6 |
| 155 | Abamectin | Macrolide | 71751-41-2 | C_48_H_72_O_14_ | [M-H]^-^ | 871.4849 | 20.4 |

Table S2 List of 195 authentic samples’ information in the study

| No. | Animal Species | Sample Source | Analyte | Content (μg/kg) | Analyte | Content (μg/kg) |
| --- | --- | --- | --- | --- | --- | --- |
| 1 | pork | abattoir | Hydrocortisone | 1.95 |  |  |
| 2 | pork | abattoir | Hydrocortisone | 2.38 | Cortisone | 1.88 |
| 3 | pork | abattoir |  |  |  |  |
| 4 | pork | abattoir | Hydrocortisone | 2.06 |  |  |
| 5 | pork | abattoir | Hydrocortisone | 3.18 |  |  |
| 6 | pork | farmers market | Hydrocortisone | 2.13 |  |  |
| 7 | pork | farmers market | Hydrocortisone | 1.27 |  |  |
| 8 | pork | farmers market | Hydrocortisone | 5.1 | Tilmicosin | 1.11 |
| 9 | pork | farmers market | Hydrocortisone | 3.55 |  |  |
| 10 | pork | farmers market | Hydrocortisone | 7.45 | Chlortetracycline | 8.96 |
| 11 | pork | supermarket | Hydrocortisone | 13.5 |  |  |
| 12 | pork | supermarket | Hydrocortisone | 8.29 | Chlortetracycline | 10.8 |
| 13 | pork | supermarket | Hydrocortisone | 3.76 |  |  |
| 14 | pork | supermarket | Hydrocortisone | 7.74 |  |  |
| 15 | pork | supermarket | Hydrocortisone | 1.77 |  |  |
| 16 | pork | supermarket | Hydrocortisone | 10.8 |  |  |
| 17 | pork | supermarket | Hydrocortisone | 7.53 |  |  |
| 18 | pork | supermarket | Hydrocortisone | 15.6 | Chlortetracycline | 11.2 |
|  |  |  | 4-Epi-Chlortetracycline | 7.33 |  |  |
| 19 | pork | supermarket | Hydrocortisone | 11 | 4-Epi-Chlortetracycline | 6.12 |
| 20 | pork | supermarket | Hydrocortisone | 5.73 | Chlortetracycline | 10.4 |
| 21 | pork | supermarket | Hydrocortisone | 2.24 |  |  |
| 22 | pork | supermarket | Hydrocortisone | 2.92 | Tilmicosin | 1.34 |
| 23 | pork | supermarket | Hydrocortisone | 4.73 |  |  |
| 24 | pork | supermarket | Hydrocortisone | 8.95 | Cortisone | 4.13 |
| 25 | pork | supermarket | Hydrocortisone | 8.39 |  |  |
| 26 | pork | supermarket | Hydrocortisone | 4.64 | Tilmicosin | 11.5 |
| 27 | pork | supermarket | Hydrocortisone | 8.47 | Tilmicosin | 1.59 |
| 28 | pork | supermarket | Hydrocortisone | 9.15 | Cortisone | 3.25 |
| 29 | pork | supermarket | Hydrocortisone | 4.95 |  |  |
| 30 | pork | supermarket | Hydrocortisone | 5.53 |  |  |
| 31 | pork | wholesale market | Hydrocortisone | 3.73 |  |  |
| 32 | pork | wholesale market | Hydrocortisone | 6.9 | Cortisone | 1.19 |
|  |  |  | Chlortetracycline | 5.08 |  |  |
| 33 | pork | wholesale market | Hydrocortisone | 13.4 | Cortisone | 2.06 |
| 34 | pork | wholesale market | Hydrocortisone | 12.1 | Thiabendazole | 0.571 |
| 35 | pork | wholesale market | Hydrocortisone | 7.74 |  |  |
| 36 | pork | wholesale market | Hydrocortisone | 24.7 | Atropine | 1.91 |
|  |  |  | 4-Epi-Chlortetracycline | 6.56 |  |  |
| 37 | pork | wholesale market | Hydrocortisone | 4.5 | Levamisole | 5.9 |
| 38 | beef | abattoir | Hydrocortisone | 2.11 |  |  |
| 39 | beef | abattoir | Hydrocortisone | 3.62 |  |  |
| 40 | beef | abattoir |  |  |  |  |
| 41 | beef | abattoir | Hydrocortisone | 2.71 | Cortisone | 1.07 |
| 42 | beef | abattoir |  |  |  |  |
| 43 | beef | abattoir |  |  |  |  |
| 44 | beef | abattoir | Hydrocortisone | 2.72 |  |  |
| 45 | beef | abattoir | Hydrocortisone | 4.06 |  |  |
| 46 | beef | farmers market | Hydrocortisone | 2.55 | Atropine | 2.45 |
| 47 | beef | farmers market | Hydrocortisone | 1.8 | Thiabendazole | 0.536 |
| 48 | beef | farmers market | Hydrocortisone | 5.08 | Cortisone | 1.45 |
|  |  |  | Chlortetracycline | 339 | Tetracycline | 14.2 |
|  |  |  | 4-Epi-Chlortetracycline | 157 |  |  |
| 49 | beef | farmers market | Hydrocortisone | 11.8 | Clenbuterol | 1.05 |
| 50 | beef | farmers market | Hydrocortisone | 7.22 | Florfenicol | 51.8 |
|  |  |  | Doxycycline | 171 | Trimethoprim | 5.08 |
|  |  |  | Enrofloxacin | 13.8 |  |  |
| 51 | beef | retail store | Hydrocortisone | 1.36 |  |  |
| 52 | beef | supermarket | Hydrocortisone | 1.92 | Testosterone | 1.07 |
| 53 | beef | supermarket | Hydrocortisone | 41.4 | Cortisone | 3.28 |
| 54 | beef | supermarket | Hydrocortisone | 3.66 |  |  |
| 55 | beef | supermarket | Sulfisomidine | 21.6 | Trimethoprim | 1.12 |
| 56 | beef | supermarket | Hydrocortisone | 1.56 |  |  |
| 57 | beef | supermarket | Hydrocortisone | 3.51 |  |  |
| 58 | beef | supermarket | Hydrocortisone | 8.56 | Ciprofloxacin | 4.18 |
|  |  |  | Enrofloxacin | 9.07 |  |  |
| 59 | beef | supermarket | Hydrocortisone | 9.2 | Chlortetracycline | 5.66 |
| 60 | beef | supermarket | Hydrocortisone | 3.91 |  |  |
| 61 | beef | supermarket | Hydrocortisone | 10.1 | Cortisone | 2.23 |
| 62 | beef | supermarket | Hydrocortisone | 2.19 |  |  |
| 63 | beef | supermarket | Hydrocortisone | 2.75 | Atropine | 1.82 |
| 64 | beef | supermarket | Hydrocortisone | 5.49 |  |  |
| 65 | beef | supermarket | Hydrocortisone | 4.52 |  |  |
| 66 | beef | supermarket | Hydrocortisone | 2.18 |  |  |
| 67 | beef | supermarket | Hydrocortisone | 2.28 |  |  |
| 68 | beef | supermarket | Hydrocortisone | 1.85 |  |  |
| 69 | beef | supermarket | Hydrocortisone | 1.38 | Atropine | 2.01 |
| 70 | beef | supermarket | Hydrocortisone | 1.96 |  |  |
| 71 | beef | supermarket | Hydrocortisone | 6.12 |  |  |
| 72 | beef | supermarket | Hydrocortisone | 10.18 | Cortisone | 2.56 |
| 73 | beef | wholesale market | Hydrocortisone | 45.4 |  |  |
| 74 | beef | wholesale market |  |  |  |  |
| 75 | beef | wholesale market | Hydrocortisone | 8.99 | Cortisone | 2.16 |
| 76 | beef | wholesale market | Hydrocortisone | 4.57 |  |  |
| 77 | beef | wholesale market |  |  |  |  |
| 78 | mutton | abattoir |  |  |  |  |
| 79 | mutton | abattoir | Hydrocortisone | 2.03 | Cortisone | 1.58 |
| 80 | mutton | abattoir |  |  |  |  |
| 81 | mutton | abattoir | Hydrocortisone | 3.11 |  |  |
| 82 | mutton | farmers market | Hydrocortisone | 2.18 |  |  |
| 83 | mutton | farmers market | Hydrocortisone | 1.14 | 4-Epi-Chlortetracycline | 7.29 |
| 84 | mutton | farmers market | Hydrocortisone | 2.47 | Cortisone | 1.89 |
|  |  |  | Chlortetracycline | 9.9 |  |  |
| 85 | mutton | farmers market | Hydrocortisone | 1.22 | 4-Epi-Chlortetracycline | 56.2 |
|  |  |  | Chlortetracycline | 103 |  |  |
| 86 | mutton | farmers market | Hydrocortisone | 4.89 | Cortisone | 2.13 |
| 87 | mutton | farmers market | Hydrocortisone | 1.42 | Chlortetracycline | 11.3 |
|  |  |  | 4-Epi-Chlortetracycline | 7.63 |  |  |
| 88 | mutton | farmers market | Chlortetracycline | 11.7 | 4-Epi-Chlortetracycline | 8.42 |
| 89 | mutton | farmers market | Hydrocortisone | 1.37 | Chlortetracycline | 6.48 |
| 90 | mutton | farmers market | Hydrocortisone | 3.14 | Enrofloxacin | 4.06 |
|  |  |  | Doxycycline | 132 | Trimethoprim | 1.79 |
| 91 | mutton | retail store | Hydrocortisone | 1.93 | Clenbuterol | 5.62 |
| 92 | mutton | retail store | Hydrocortisone | 1.5 | Chlortetracycline | 20.1 |
|  |  |  | 4-Epi-Chlortetracycline | 15.5 |  |  |
| 93 | mutton | supermarket | Hydrocortisone | 2.33 | Enrofloxacin | 3.88 |
| 94 | mutton | supermarket | Hydrocortisone | 3.21 |  |  |
| 95 | mutton | supermarket | Hydrocortisone | 5.06 |  |  |
| 96 | mutton | supermarket | Hydrocortisone | 2.63 | Chlortetracycline | 5.4 |
| 97 | mutton | supermarket | Hydrocortisone | 1.65 |  |  |
| 98 | mutton | supermarket | Hydrocortisone | 13.5 |  |  |
| 99 | mutton | supermarket | Hydrocortisone | 1.62 |  |  |
| 100 | mutton | supermarket | Hydrocortisone | 1.45 |  |  |
| 101 | mutton | supermarket | Hydrocortisone | 1.91 |  |  |
| 102 | mutton | supermarket | Hydrocortisone | 3.51 |  |  |
| 103 | mutton | wholesale market | Hydrocortisone | 6.27 |  |  |
| 104 | mutton | wholesale market | Hydrocortisone | 5.37 |  |  |
| 105 | mutton | wholesale market | Hydrocortisone | 4.827 |  |  |
| 106 | mutton | wholesale market | Hydrocortisone | 2.46 |  |  |
| 107 | mutton | wholesale market | Hydrocortisone | 1.47 | Chlortetracycline | 7.03 |
|  |  |  | 4-Epi-Chlortetracycline | 5.7 |  |  |
| 108 | mutton | wholesale market | Hydrocortisone | 5.8 |  |  |
| 109 | mutton | wholesale market | Hydrocortisone | 2.4 |  |  |
| 110 | mutton | wholesale market | Hydrocortisone | 4.45 |  |  |
| 111 | mutton | wholesale market | Chlortetracycline | 8.6 | 4-Epi-Chlortetracycline | 6.39 |
| 112 | mutton | wholesale market | Hydrocortisone | 1.61 |  |  |
| 113 | mutton | wholesale market | Hydrocortisone | 1.59 | Chlortetracycline | 6.72 |
| 114 | pork liver | abattoir |  |  |  |  |
| 115 | pork liver | abattoir | Enrofloxacin | 4.22 |  |  |
| 116 | pork liver | abattoir |  |  |  |  |
| 117 | pork liver | abattoir |  |  |  |  |
| 118 | pork liver | farmers market |  |  |  |  |
| 119 | pork liver | farmers market | Chlortetracycline | 29.5 | 4-Epi-Chlortetracycline | 26.3 |
| 120 | pork liver | farmers market |  |  |  |  |
| 121 | pork liver | farmers market | Enrofloxacin | 3.75 |  |  |
| 122 | pork liver | farmers market | 4-Epi-Chlortetracycline | 11.7 |  |  |
| 123 | pork liver | farmers market | Chlortetracycline | 7.66 | Tilmicosin | 4.92 |
|  |  |  | 4-Epi-Chlortetracycline | 8.79 |  |  |
| 124 | pork liver | farmers market | Chlortetracycline | 6.21 |  |  |
| 125 | pork liver | farmers market | Doxycycline | 29.6 | 4-Epi-Chlortetracycline | 11.2 |
| 126 | pork liver | farmers market |  |  |  |  |
| 127 | pork liver | farmers market | Ofloxacin | 225 | Chlortetracycline | 51.3 |
|  |  |  | 4-Epi-Chlortetracycline | 21.3 | Doxycycline | 20.7 |
|  |  |  | Lincomycin | 4.03 | Trimethoprim | 1.2 |
| 128 | pork liver | retail store | Chlortetracycline | 9.47 |  |  |
| 129 | pork liver | retail store | Olaquindox | 19 |  |  |
| 130 | pork liver | retail store |  |  |  |  |
| 131 | pork liver | supermarket | Chlortetracycline | 147 | 4-Epi-Chlortetracycline | 103 |
| 132 | pork liver | supermarket | Olaquindox | 5.37 |  |  |
| 133 | pork liver | supermarket |  |  |  |  |
| 134 | pork liver | supermarket | Lignocaine | 0.505 |  |  |
| 135 | pork liver | supermarket |  |  |  |  |
| 136 | pork liver | supermarket | Chlortetracycline | 8.23 | Tilmicosin | 2.49 |
| 137 | pork liver | wholesale market | Enrofloxacin | 8.05 | Doxycycline | 8.46 |
| 138 | pork liver | wholesale market |  |  |  |  |
| 139 | pork liver | wholesale market | Tilmicosin | 98.7 |  |  |
| 140 | pork liver | wholesale market | Tilmicosin | 11.4 | Enrofloxacin | 7.71 |
| 141 | pork liver | wholesale market | Chlortetracycline | 63.8 |  |  |
| 142 | pork liver | wholesale market | Tilmicosin | 177 |  |  |
| 143 | pork liver | wholesale market | Enrofloxacin | 7.82 |  |  |
| 144 | pork liver | wholesale market |  |  |  |  |
| 145 | pork liver | wholesale market | Chlortetracycline | 58.1 | 4-Epi-Chlortetracycline | 39.5 |
|  |  |  | Olaquindox | 1.65 |  |  |
| 146 | pork liver | wholesale market | Olaquindox | 1.93 |  |  |
| 147 | pork liver | wholesale market |  |  |  |  |
| 148 | pork liver | wholesale market | Chlortetracycline | 10.1 | 4-Epi-Chlortetracycline | 8 |
| 149 | pork liver | wholesale market | Chlortetracycline | 21.3 | 4-Epi-Chlortetracycline | 16.7 |
| 150 | chicken | abattoir |  |  |  |  |
| 151 | chicken | abattoir |  |  |  |  |
| 152 | chicken | abattoir |  |  |  |  |
| 153 | chicken | abattoir |  |  |  |  |
| 154 | chicken | abattoir |  |  |  |  |
| 155 | chicken | abattoir |  |  |  |  |
| 156 | chicken | abattoir |  |  |  |  |
| 157 | chicken | abattoir |  |  |  |  |
| 158 | chicken | farmers market | Thiabendazole | 1.14 |  |  |
| 159 | chicken | retail store | Oxytetracycline | 11.4 |  |  |
| 160 | chicken | supermarket |  |  |  |  |
| 161 | chicken | supermarket |  |  |  |  |
| 162 | chicken | supermarket |  |  |  |  |
| 163 | chicken | supermarket | Thiabendazole | 0.635 |  |  |
| 164 | chicken | supermarket |  |  |  |  |
| 165 | chicken | supermarket |  |  |  |  |
| 166 | chicken | supermarket | Enrofloxacin | 4.27 |  |  |
| 167 | chicken | supermarket |  |  |  |  |
| 168 | chicken | supermarket |  |  |  |  |
| 169 | chicken | supermarket | Diclazuril | 26 |  |  |
| 170 | chicken | supermarket |  |  |  |  |
| 171 | chicken | supermarket |  |  |  |  |
| 172 | chicken | supermarket |  |  |  |  |
| 173 | chicken | supermarket |  |  |  |  |
| 174 | chicken liver | abattoir |  |  |  |  |
| 175 | chicken liver | abattoir | Tilmicosin | 1.45 |  |  |
| 176 | chicken liver | abattoir |  |  |  |  |
| 177 | chicken liver | abattoir |  |  |  |  |
| 178 | chicken liver | abattoir |  |  |  |  |
| 179 | chicken liver | abattoir |  |  |  |  |
| 180 | chicken liver | farmers market |  |  |  |  |
| 181 | chicken liver | farmers market | Chlortetracycline | 10.4 | Tilmicosin | 32.1 |
|  |  |  | Amantadine | 29.7 | 4-Epi-Chlortetracycline | 6.77 |
| 182 | chicken liver | farmers market | Chlortetracycline | 29.8 | Tilmicosin | 179 |
|  |  |  | 4-Epi-Chlortetracycline | 14.3 |  |  |
| 183 | chicken liver | farmers market |  |  |  |  |
| 184 | chicken liver | farmers market | Diclazuril | 320 |  |  |
| 185 | chicken liver | farmers market |  |  |  |  |
| 186 | chicken liver | retail store |  |  |  |  |
| 187 | chicken liver | supermarket | Doxycycline | 18.1 | Tilmicosin | 167 |
| 188 | chicken liver | wholesale market | Doxycycline | 11.7 |  |  |
| 189 | chicken liver | wholesale market |  |  |  |  |
| 190 | chicken liver | wholesale market |  |  |  |  |
| 191 | chicken liver | wholesale market |  |  |  |  |
| 192 | chicken liver | wholesale market | Chlortetracycline | 31.7 | 4-Epi-Chlortetracycline | 23.2 |
| 193 | chicken liver | wholesale market |  |  |  |  |
| 194 | chicken liver | wholesale market |  |  |  |  |
| 195 | chicken liver | wholesale market |  |  |  |  |


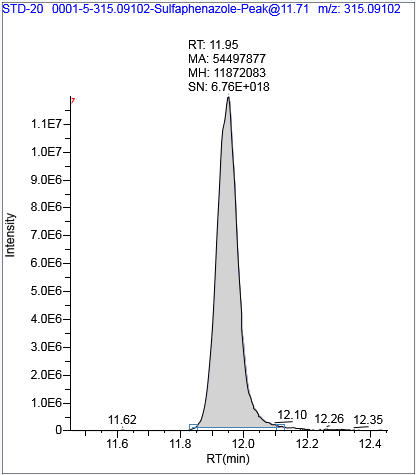

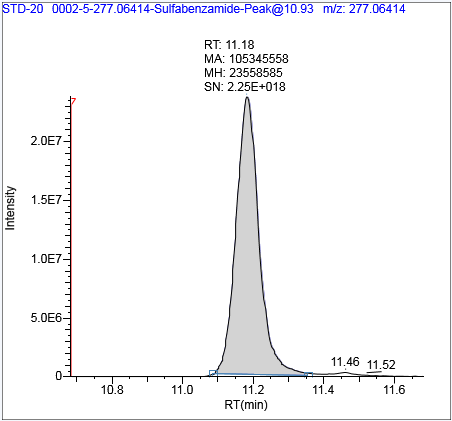

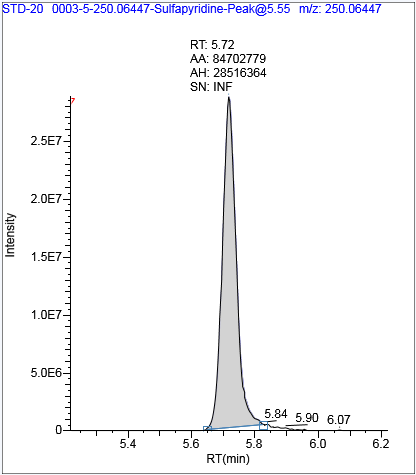


Sulfaphenazole Sulfabenzamide Sulfapyridine


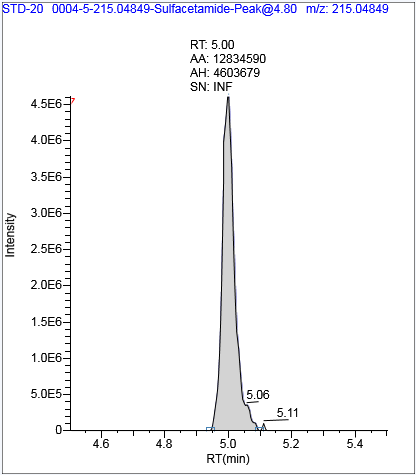

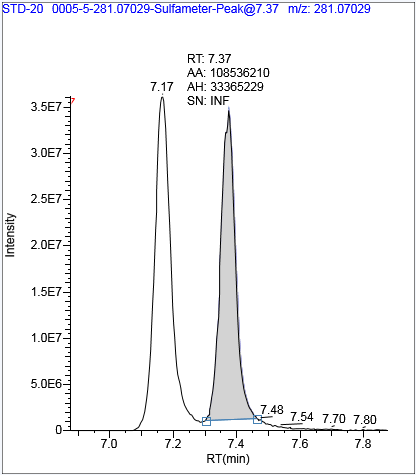

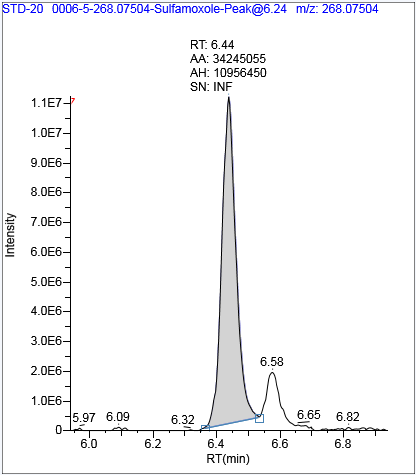


Sulfacetamide Sulfameter Sulfamoxole


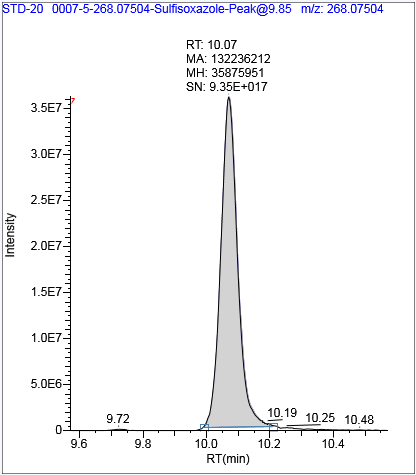

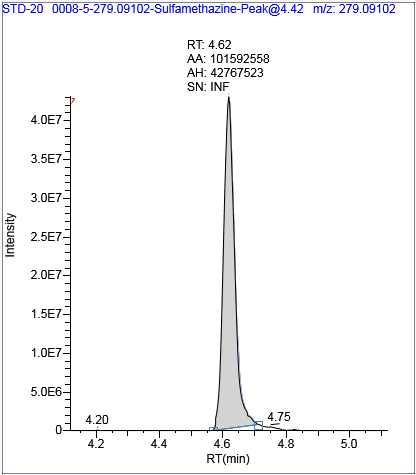

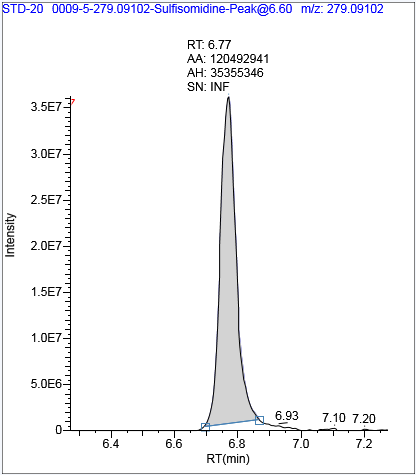


Sulfisoxazole Sulfamethazine Sulfisomidine


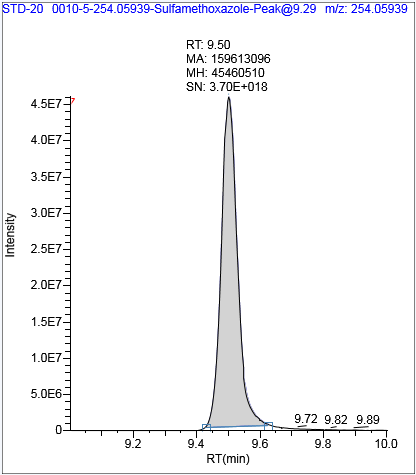

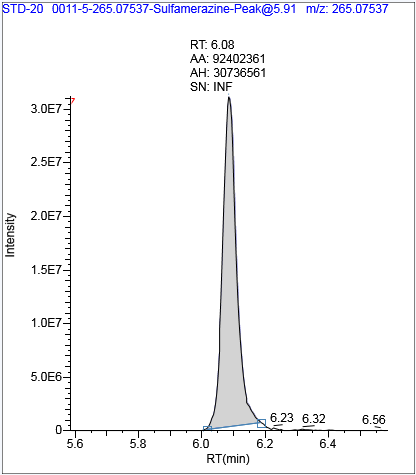

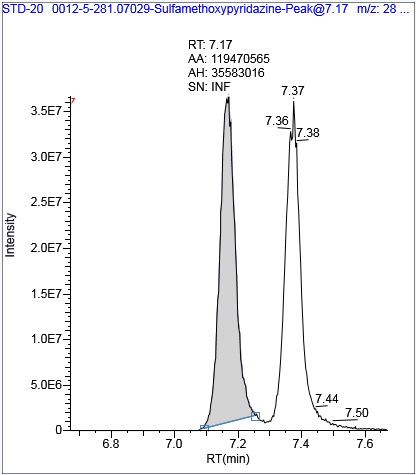


Sulfamethoxazole Sulfamerazine Sulfamethoxypyridazine


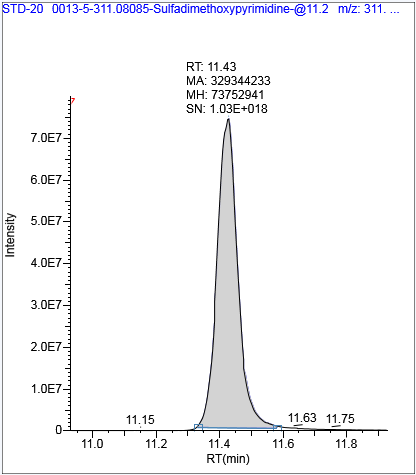

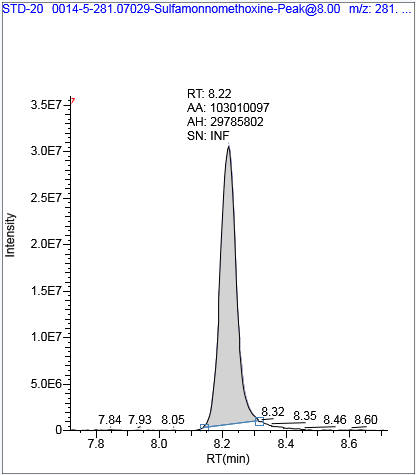

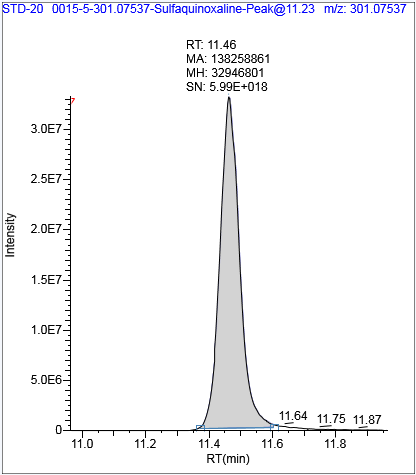


Sulfadimethoxypyrimidine Sulfamonnomethoxine Sulfaquinoxaline


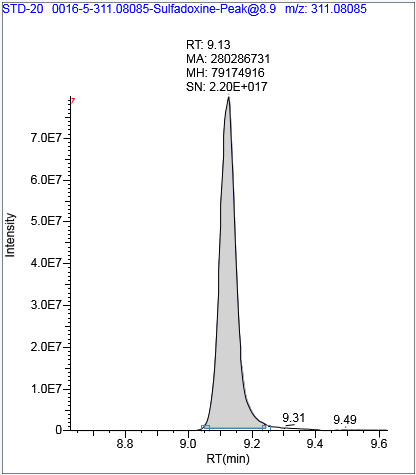

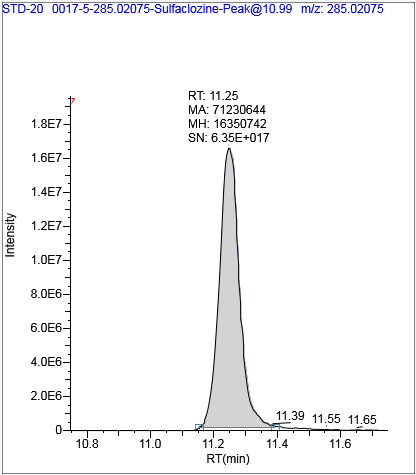

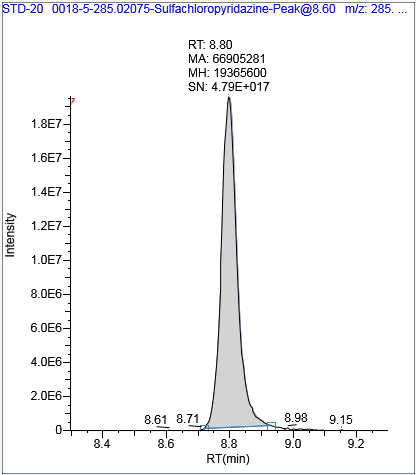


Sulfadimoxine Sulfaclozine Sulfachloropyridazine


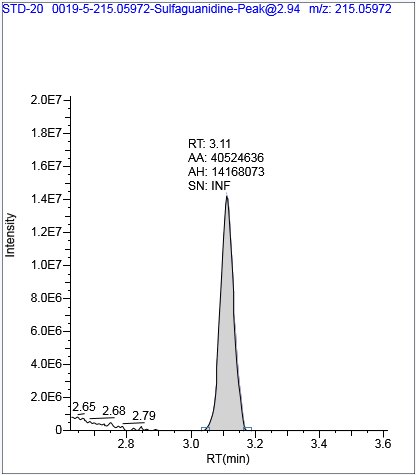

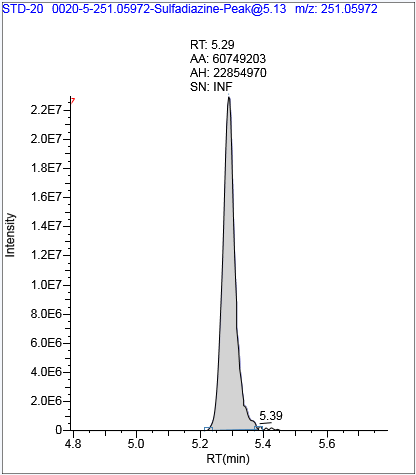

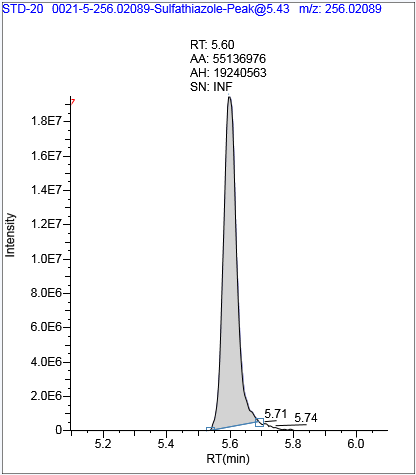


Sulfaguanidine Sulfadiazine Sulfathiazole


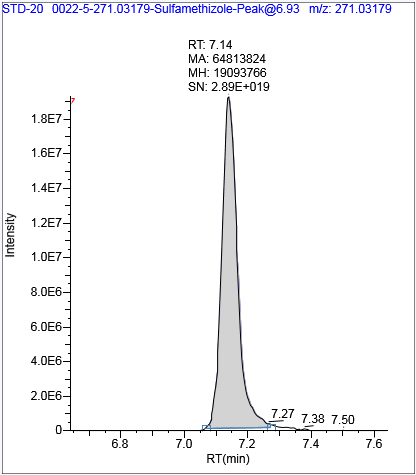

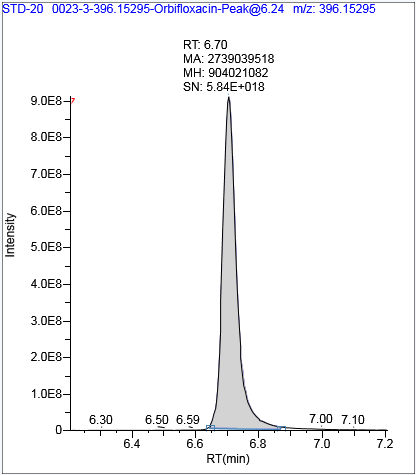

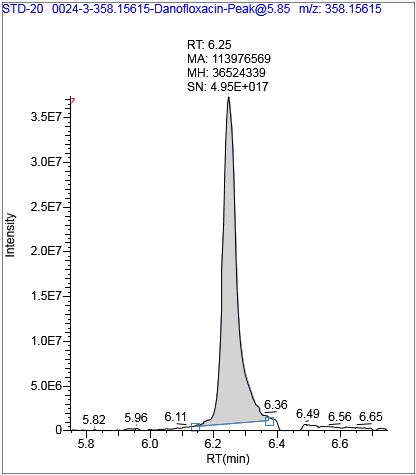


Sulfamethizole Orbifloxacin Danofloxacin


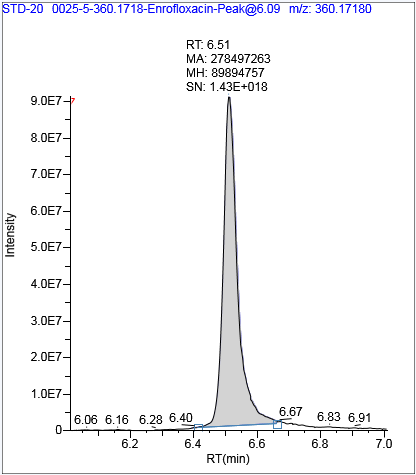

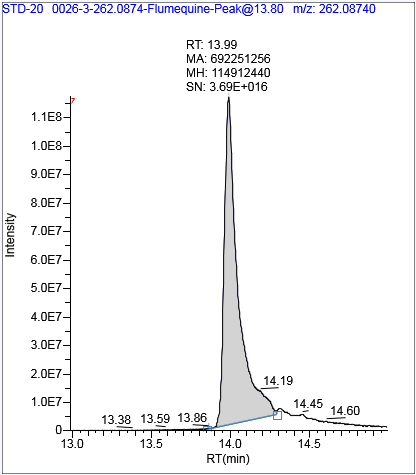

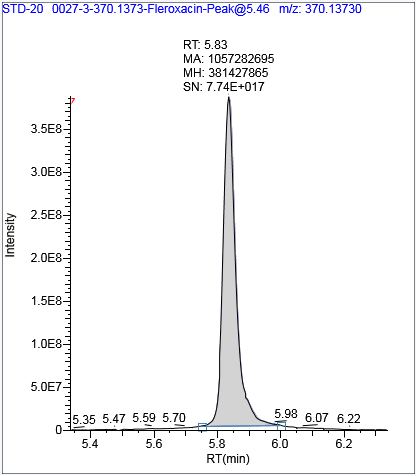


Enrofloxacin Flumequine Fleroxacin


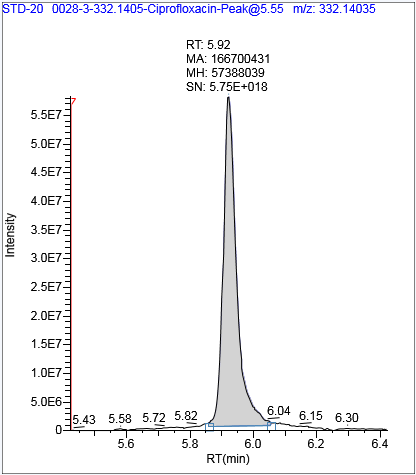

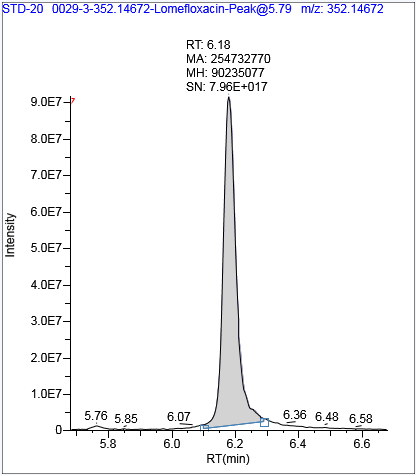

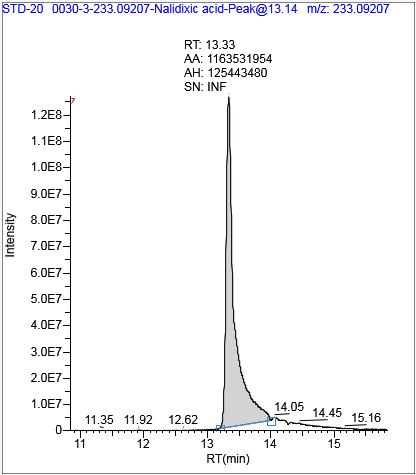


Ciprofloxacin Lomefloxacin Nalidixic acid


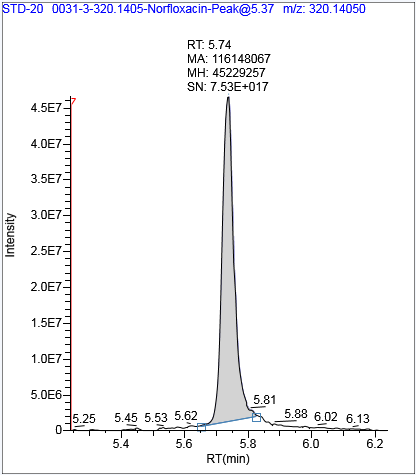

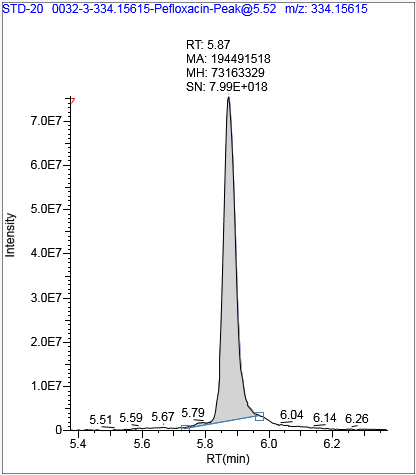

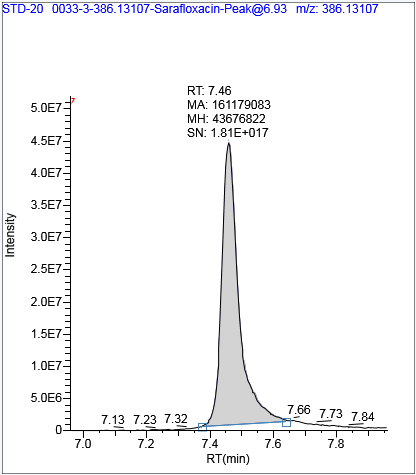


Norfloxacin Pefloxacin Sarafloxacin


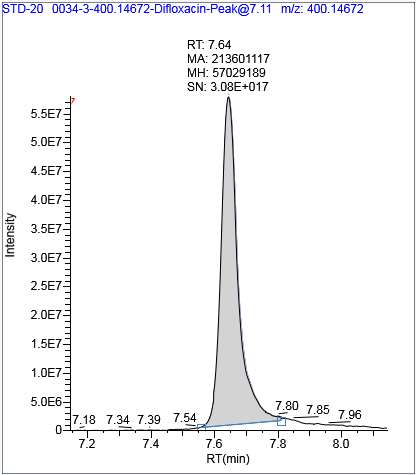

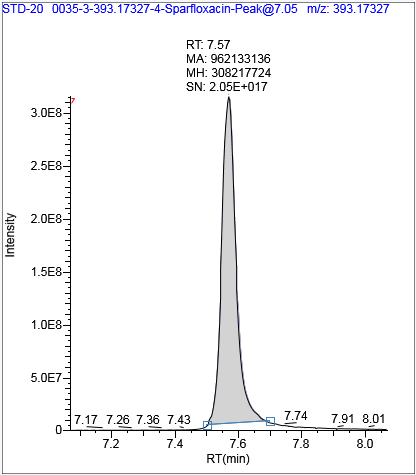

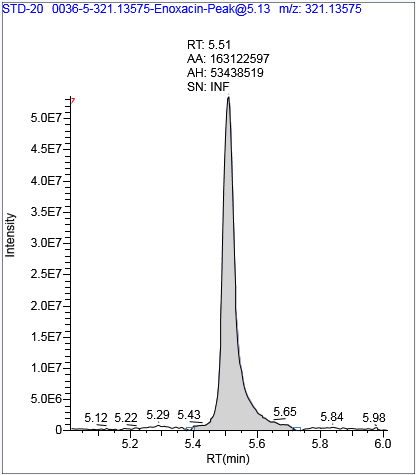


Difloxacin Sparfloxacin Enoxacin


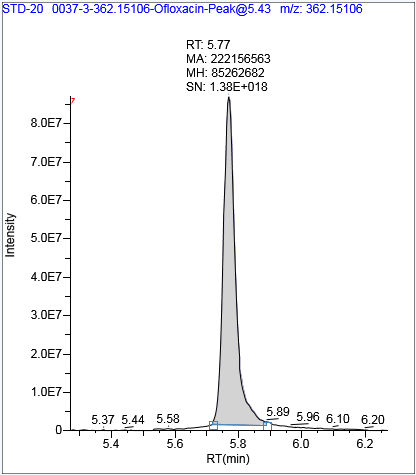

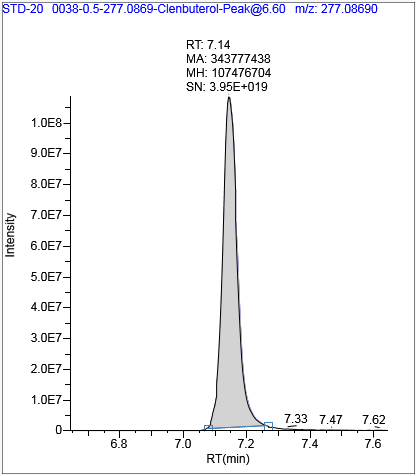

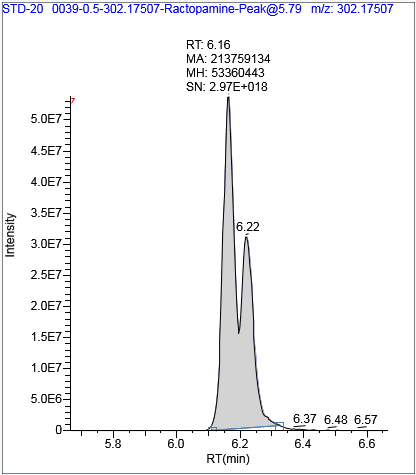


Ofloxacin Clenbuterol Ractopamine


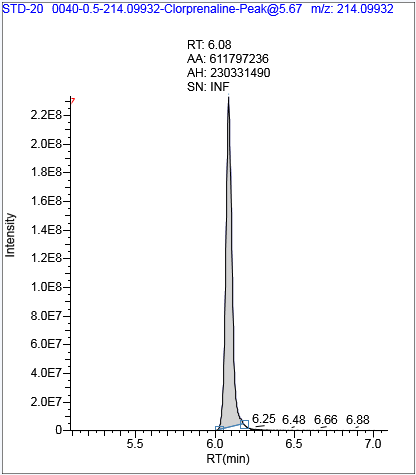

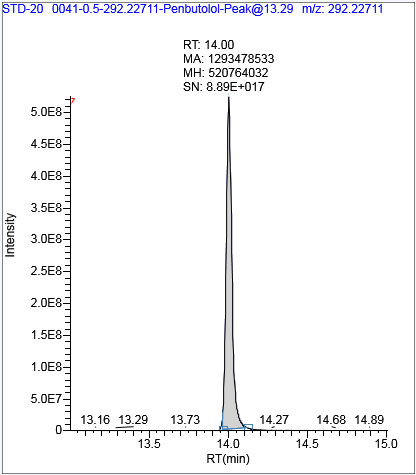

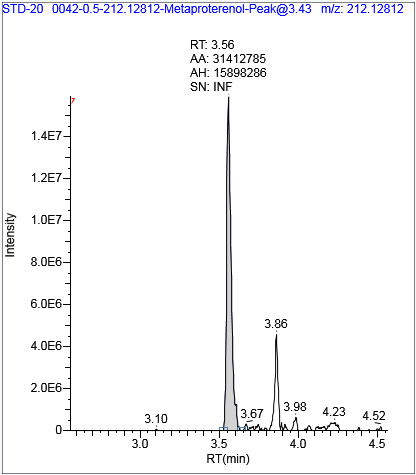


Clorprenaline Penbutolol Metaproterenol


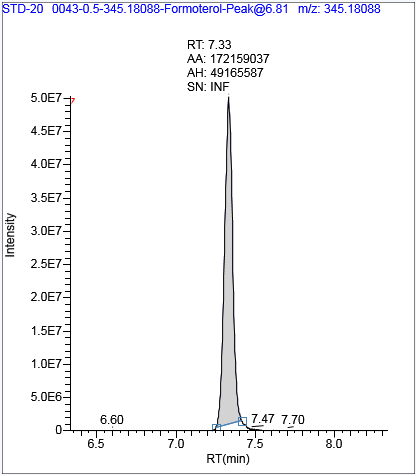

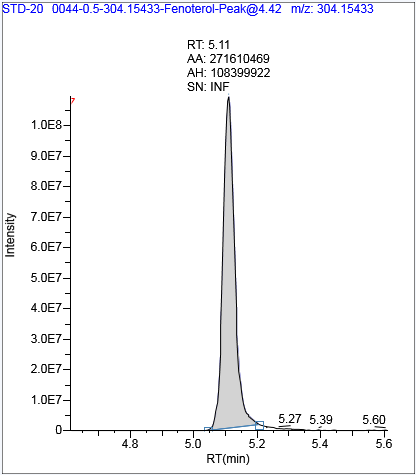

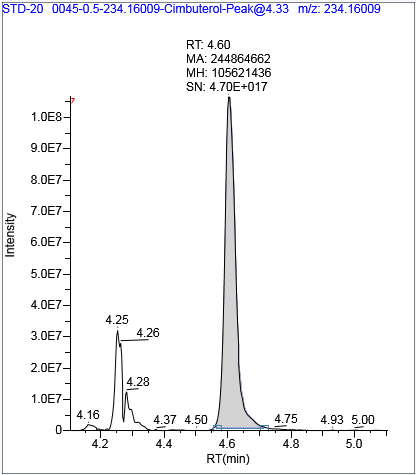


Formoterol Fenoterol Cimbuterol


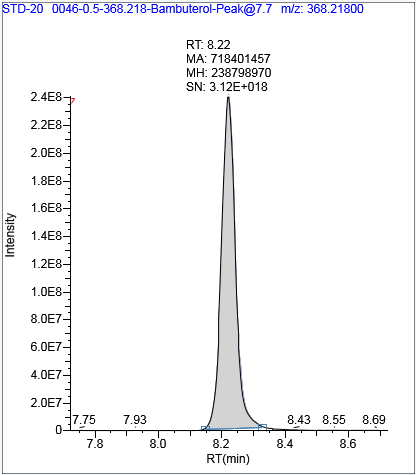

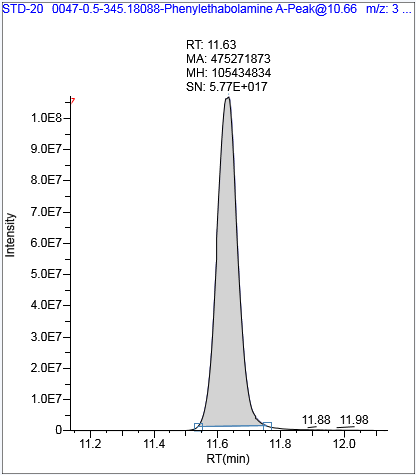

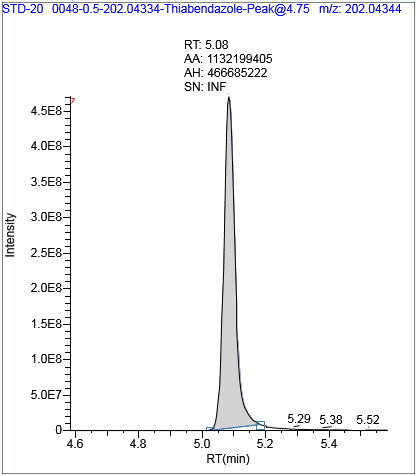


Bambuterol Phenylethanolamine A Thiabendazole


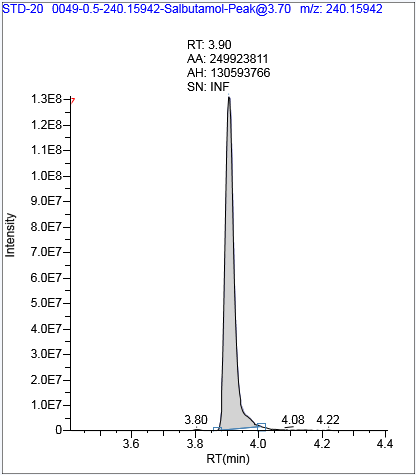

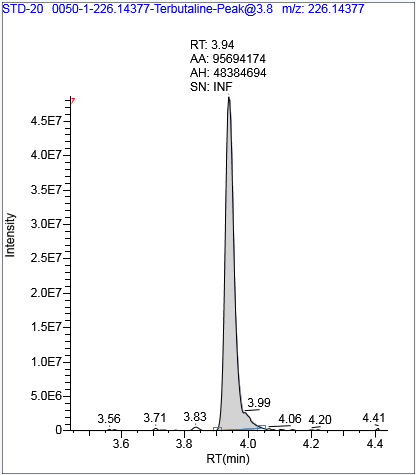

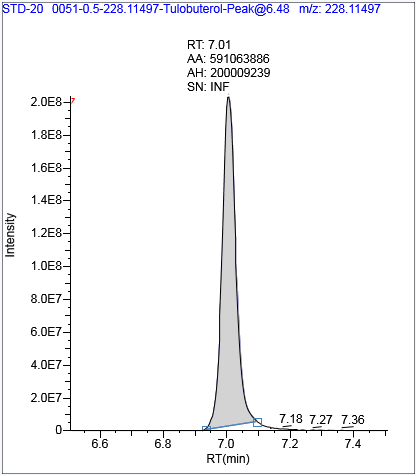


Salbutamol Terbutaline Tulobuterol


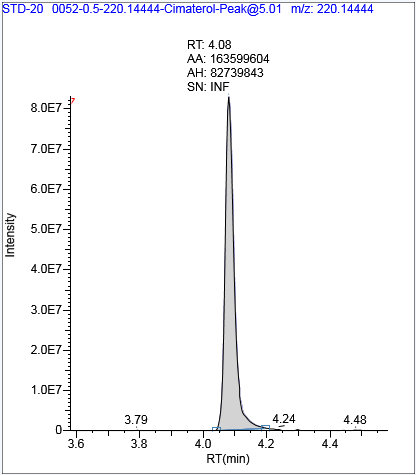

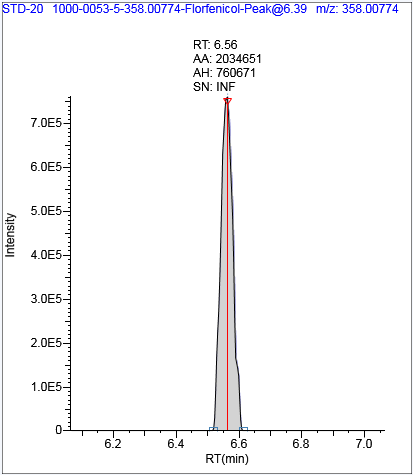

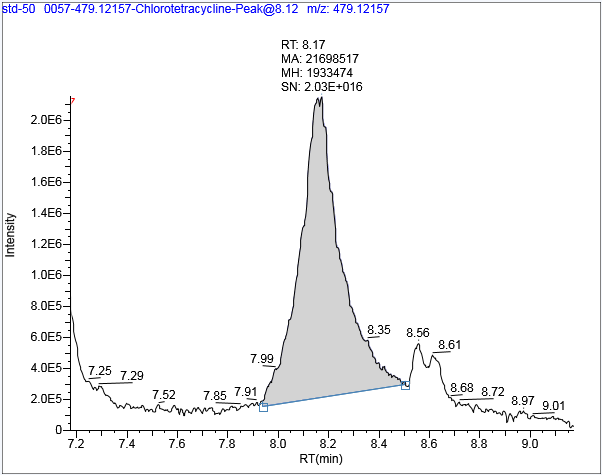


Cimaterol Florfenicol Chlorotetracycline


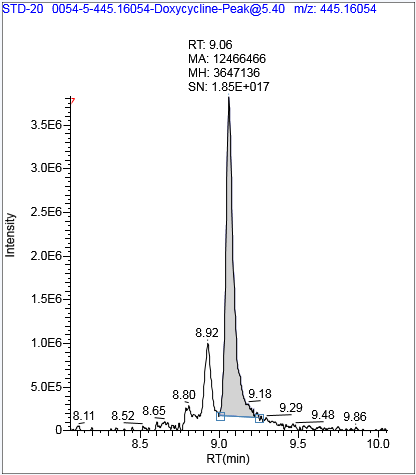

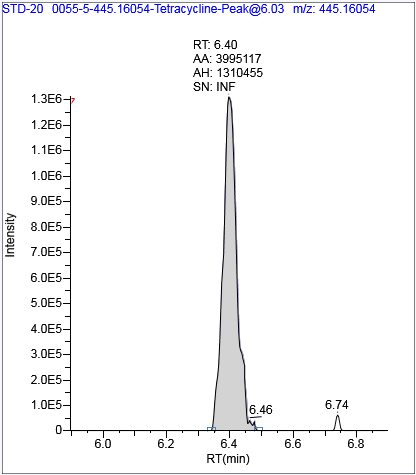

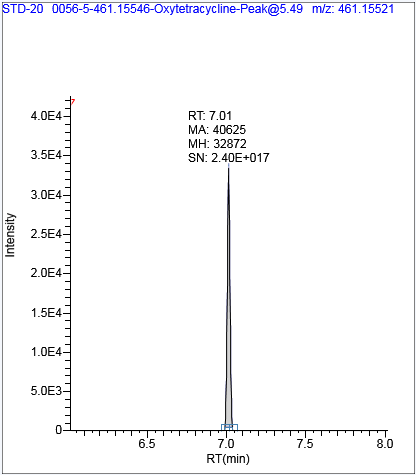


Doxycycline Tetracycline Oxytetracycline


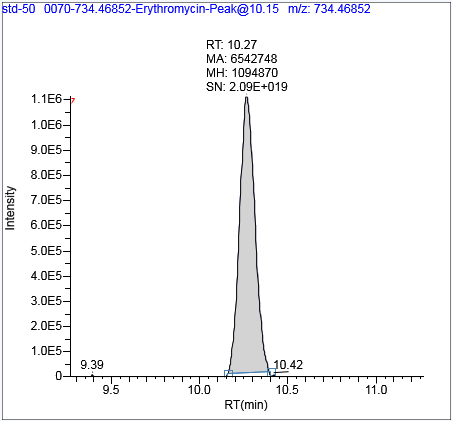

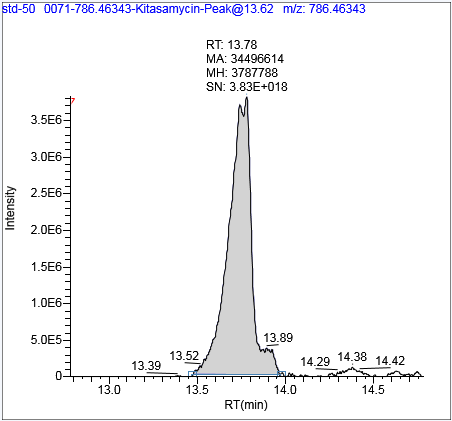

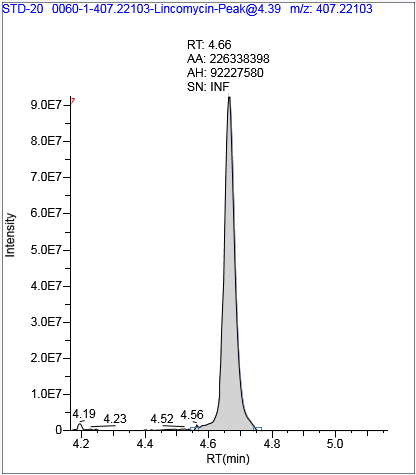


Erythromycin Kitasamycin Lincomycin


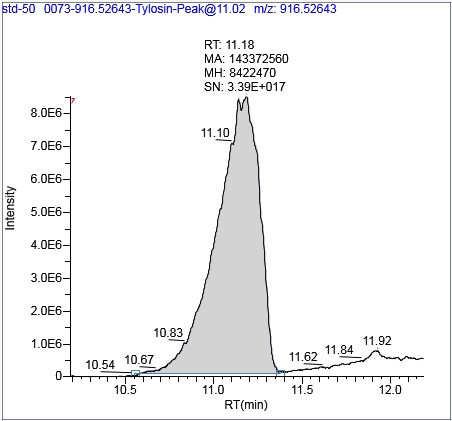

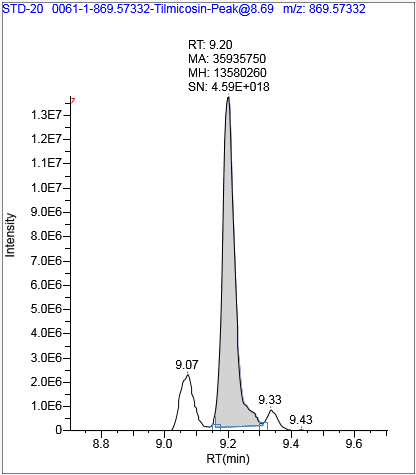

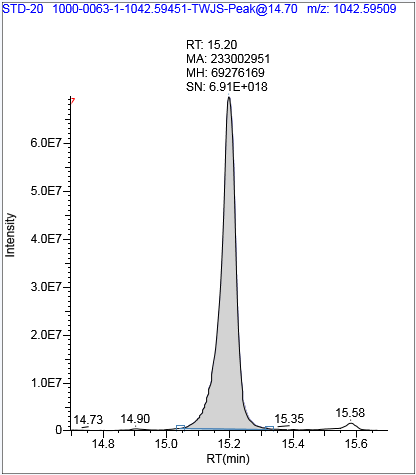


[Tylosin](javascript:showMsgDetail('ProductSynonyms.aspx?CBNumber=CB7708088&postData3=CN&SYMBOL_Type=A');) Tilmicosin Tylosin 3-acetate

4B-(3-methylbutanoate)(2R,3R)-

2,3-dihydroxybutanedioate


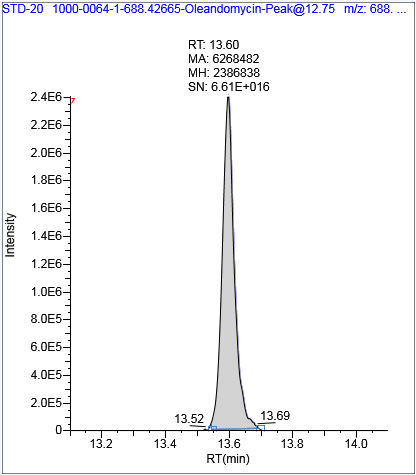

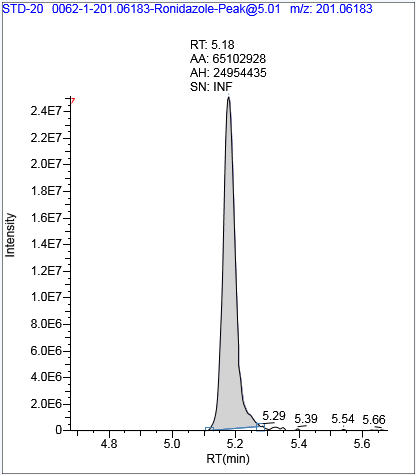

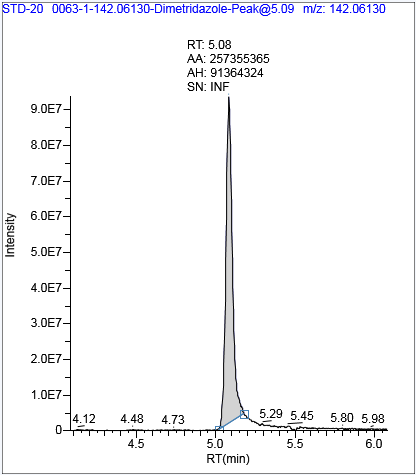


Oleandomycin Ronidazole Dimetridazole


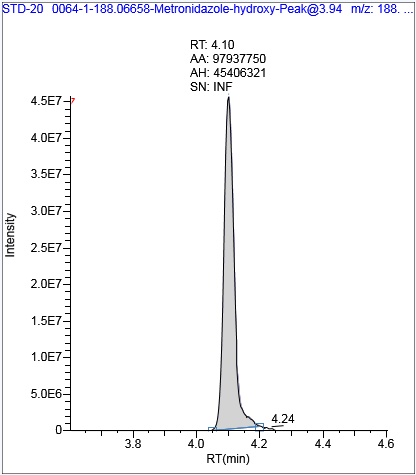

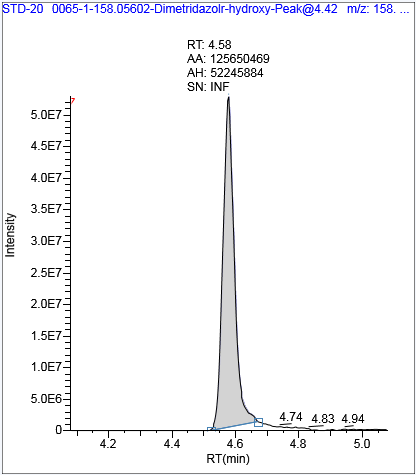

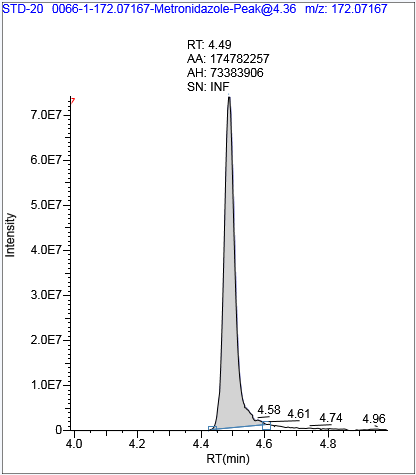


Metronidazole-hydroxy Dimetridazolr-hydroxy Metronidazole


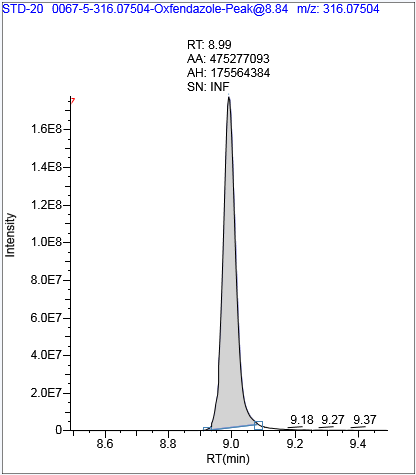

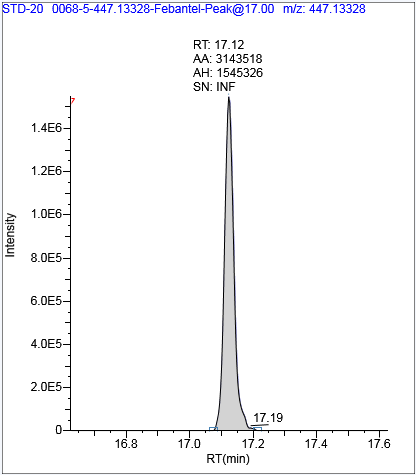

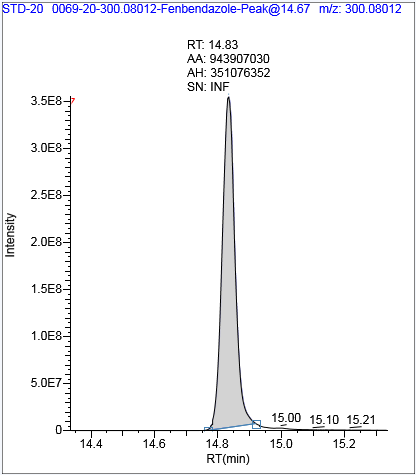


Oxfendazole Febantel Fenbendazole


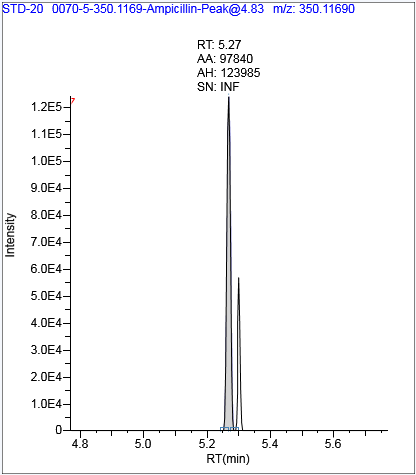

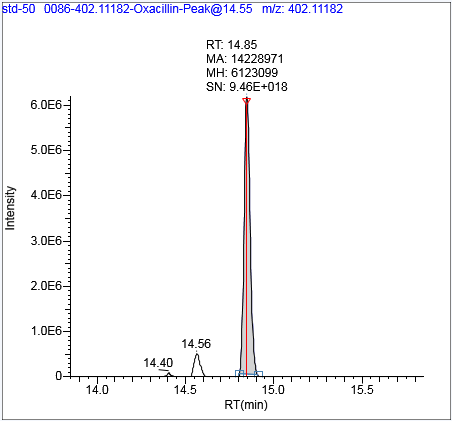

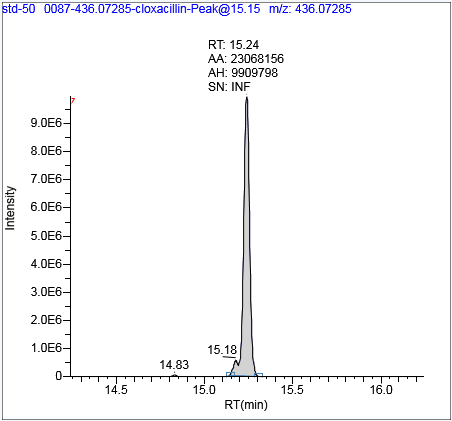


Ampicillin Oxacillin Cloxacillin


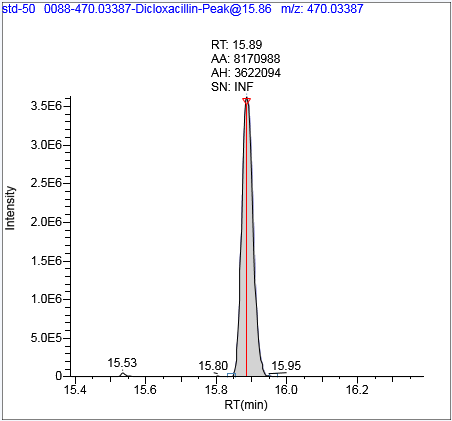

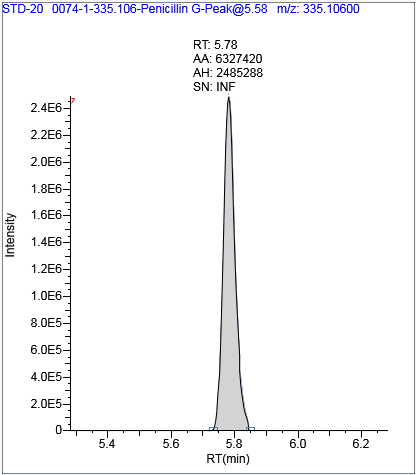

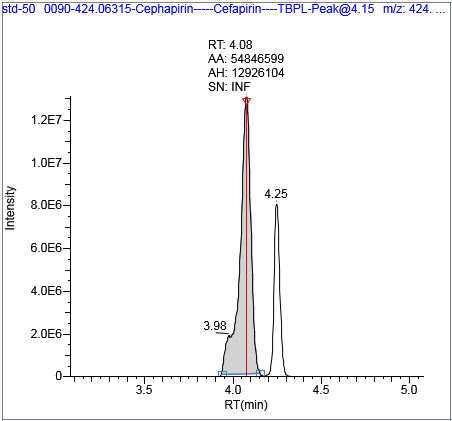


Dicloxacillin Penicillin G Cephapirin


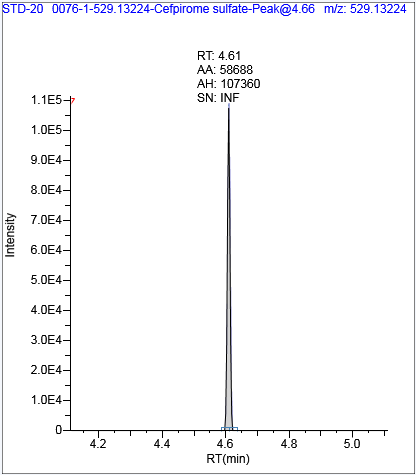

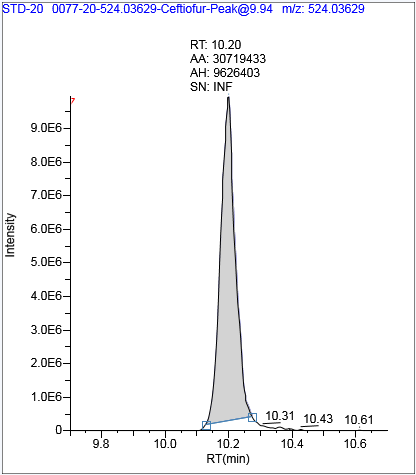

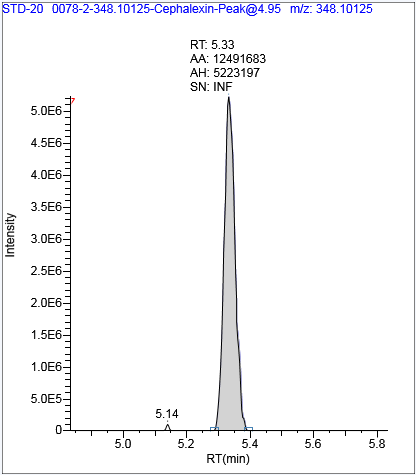


Cefpirome Ceftiofur Cephalexin


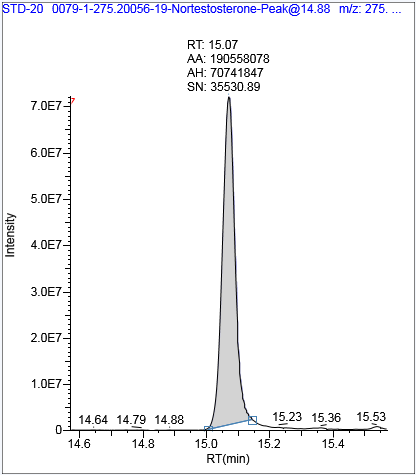

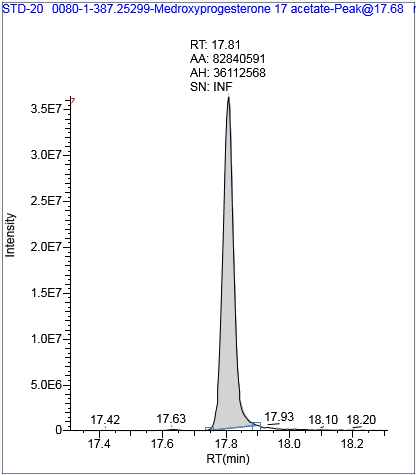

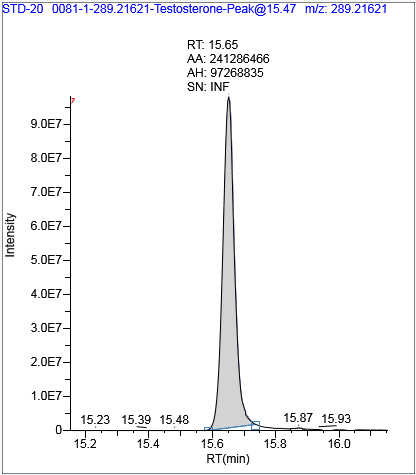


19-Nortestosterone Medroxyprogesterone Acetate Testosterone


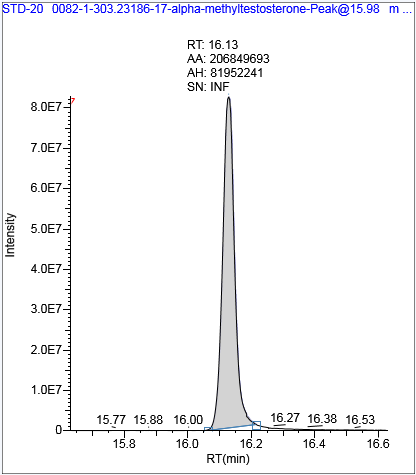

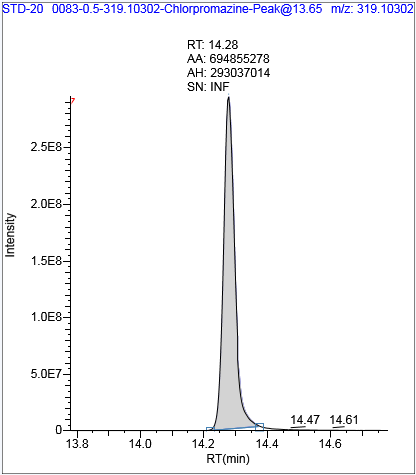

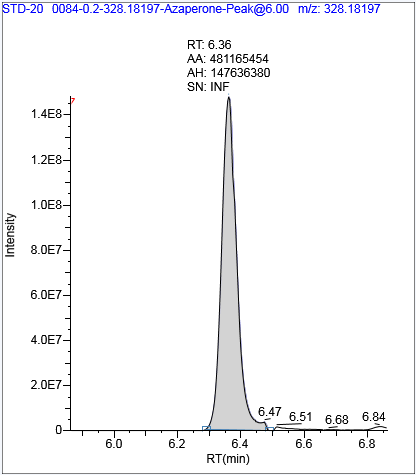


17-Methyltestosterone Chlorpromazine Azaperone


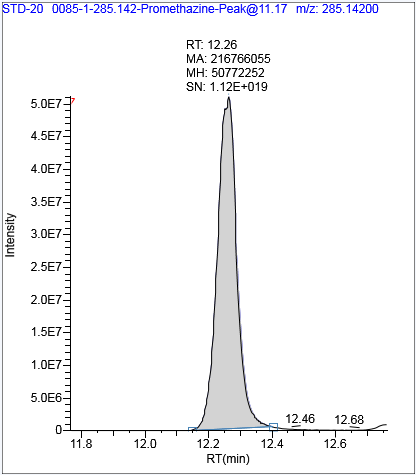

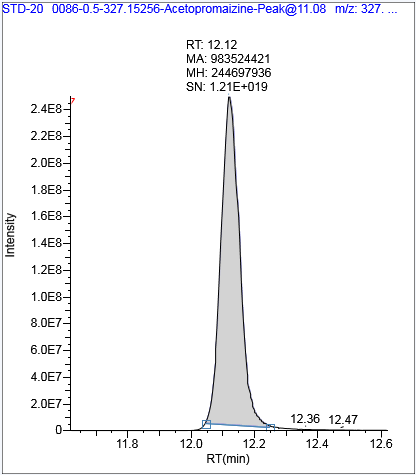

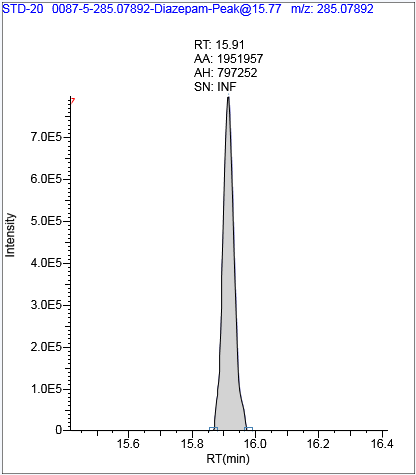


Promethazine Acetopromaizine Diazepam


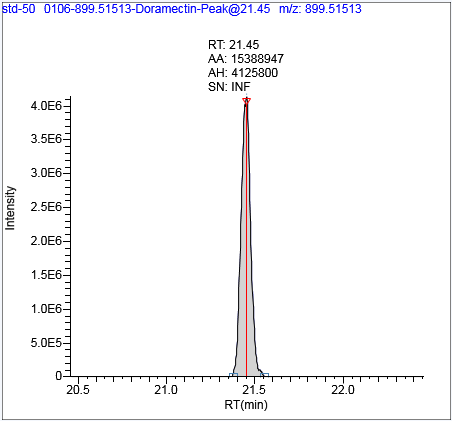

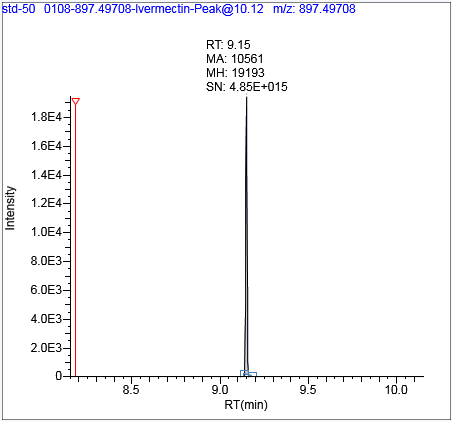

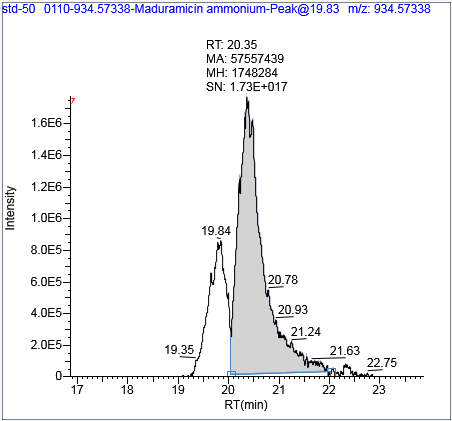


Doramectin Ivermectin [Maduramicin ammonium](javascript:showMsgDetail('ProductSynonyms.aspx?CBNumber=CB9703623&postData3=CN&SYMBOL_Type=A');)


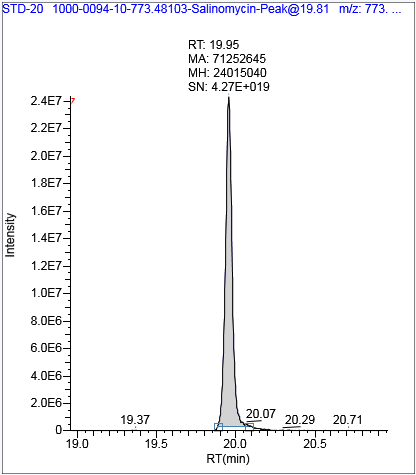

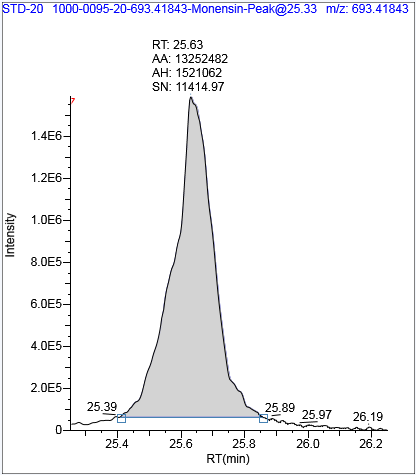

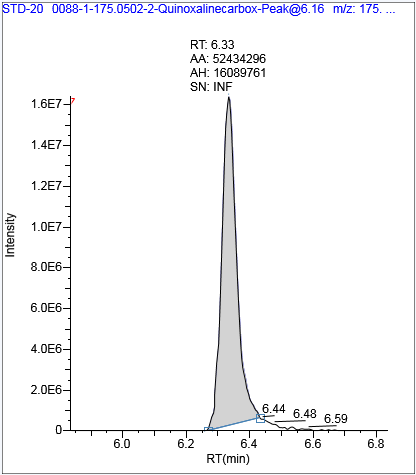


Salinomycin Monensin 2-Quinoxalinecarbox


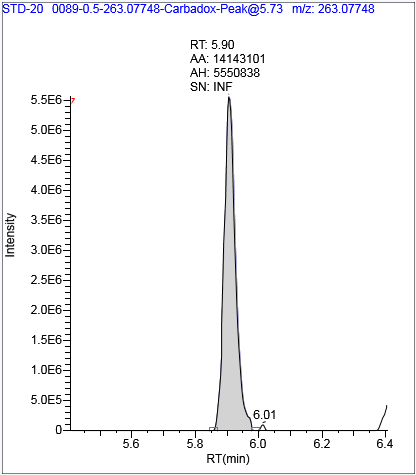

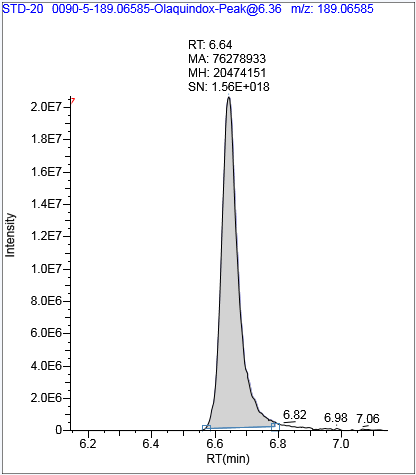

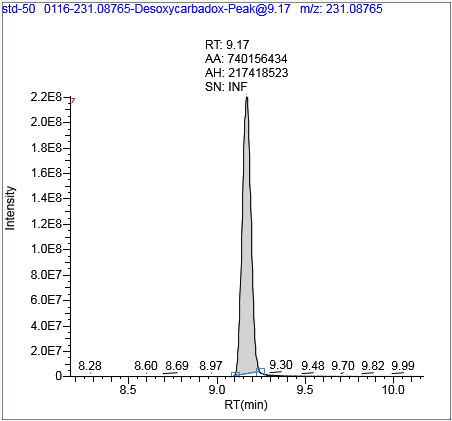


Carbadox Olaquindox Desoxycarbadox


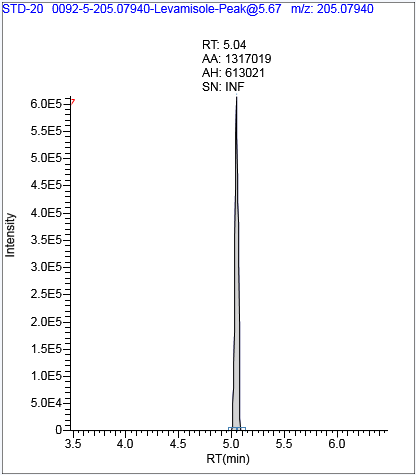


Levamisole Carbofuran Coumaphos

Fenthion-sulfone Fenthion-sulfoxide Malathion

Phoxim Dipterex Trimethoprim

Atropine Procaine Lignocaine

Scopolamine Anisodamine Sulfanilamide

Mabuterol(Ambuterol) Cefazolin Amantadine

Rimantadine Ribavirin Oseltamivir

4-Epi-Oxytetracycline 4-Epi-Chlortetracycline 4-Epi-Demeclocycline

Nequinate Clopidol Amprolium

Halofuginone hydrobromide Narasin Albendazole-2-aminosulfone

Albendazole sulfone Albendazole sulfoxide Albendazole

Diclazuril Chloramphenicol Beclomethasone

Cortisone acetate Dexamethasone Methylprednisolone

Cortisone Meprednisone Hydrocortisone

Fludrocortisone acetate Betamethasone Diethylstilbestrol

Estradiol Hexestrol Lasalocid

Fipronil Clazuril Nicarbazin

Fipronil sulfone Fipronil sulfide Fipronil desulfinyl

Thiamphenicol Abamectin

Fig. S1 Extract ion chromatogram of 155 veterinary drugs
